# Edeine B_1_ produced by *Brevibacillus brevis* reduces the virulence of a plant pathogenic fungus by inhibiting mitochondrial respiration

**DOI:** 10.1128/mbio.01351-24

**Published:** 2024-06-11

**Authors:** Bomin Kim, Minh Van Nguyen, Jiyeun Park, Yeong Seok Kim, Jae Woo Han, Joo-Youn Lee, Junhyun Jeon, Hokyoung Son, Gyung Ja Choi, Hun Kim

**Affiliations:** 1 Center for Eco-friendly New Materials, Korea Research Institute of Chemical Technology, Daejeon, South Korea; 2 Department of Medicinal Chemistry and Pharmacology, University of Science and Technology, Daejeon, South Korea; 3 Department of Agricultural Biotechnology, Seoul National University, Seoul, South Korea; 4 Therapeutics and Biotechnology Division, Korea Research Institute of Chemical Technology, Daejeon, South Korea; 5 Department of Biotechnology, Yeungnam University, Gyeongsan, South Korea; University of Nebraska–Lincoln, Lincoln, Nebraska, USA

**Keywords:** *Brevibacillus brevis*, respiratory inhibition, biocontrol, antifungal activity, *Fusarium graminearum*

## Abstract

Plant pathogenic fungi cause serious diseases, which result in the loss of crop yields and reduce the quality of crops worldwide. To counteract the escalating risks of chemical fungicides, interest in biological control agents to manage plant diseases has significantly increased. In this study, we comprehensively screened microbial culture filtrates using a yeast screening system to find microbes exhibiting respiratory inhibition activity. Consequently, we found a soil-borne microbe *Brevibacillus brevis* HK544 strain exhibiting a respiration inhibitory activity and identified edeine B_1_ (EB_1_) from the culture filtrate of HK544 as the active compound of the respiration inhibition activity. Furthermore, against a plant pathogenic fungus *Fusarium graminearum*, our results showed that EB_1_ has effects on multiple aspects of respiration with the downregulation of most of the mitochondrial-related genes based on transcriptome analysis, differential EB_1_-sensitivity from targeted mutagenesis, and the synergistic effects of EB_1_ with electron transport chain complex inhibitors. With the promising plant disease control efficacy of *B. brevis* HK544 producing EB_1_, our results suggest that *B. brevis* HK544 has potential as a biocontrol agent for Fusarium head blight.

**IMPORTANCE:**

As a necrotrophic fungus, *Fusarium graminearum* is a highly destructive pathogen causing severe diseases in cereal crops and mycotoxin contamination in grains. Although chemical control is considered the primary approach to control plant disease caused by *F. graminearum*, fungicide-resistant strains have been detected in the field after long-term continuous application of fungicides. Moreover, applying chemical fungicides that trigger mycotoxin biosynthesis is a great concern for many researchers. Biocontrol of *Fusarium* head blight (FHB) by biological control agents (BCAs) represents an alternative approach and could be used as part of the integrated management of FHB and mycotoxin production. The most extensive studies on bacterial BCAs-fungal communications in agroecosystems have focused on antibiosis. Although many BCAs in agricultural ecology have already been used for fungal disease control, the molecular mechanisms of antibiotics produced by BCAs remain to be elucidated. Here, we found a potential BCA (*Brevibacillus brevis* HK544) with a strong antifungal activity based on the respiration inhibition activity with its active compound edeine B_1_ (EB_1_). Furthermore, our results showed that EB_1_ secreted by HK544 suppresses the expression of the mitochondria-related genes of *F. graminearum*, subsequently suppressing fungal development and the virulence of *F. graminearum*. In addition, EB_1_ exhibited a synergism with complex I inhibitors such as rotenone and fenazaquin. Our work extends our understanding of how *B. brevis* HK544 exhibits antifungal activity and suggests that the *B. brevis* HK544 strain could be a valuable source for developing new crop protectants to control *F. graminearum*.

## INTRODUCTION

Plant pathogenic fungi cause serious diseases, resulting in crop yield loss and reducing crop quality worldwide (1). To control plant disease, chemical fungicides have been widely used, but the overuse of these fungicides has led to concerns about the hazards to humans, animals, and the environment and the increase of fungicide resistance (2). The resistance to single‐site fungicides, including methyl benzimidazoles, demethylation inhibitors, quinone outside inhibitors (QoIs), and succinate dehydrogenase inhibitors (SDHIs), has been reported in field trials and has spread throughout fungal pathogen populations, reducing the disease control efficacy. To date, the resistance mechanisms of plant pathogenic fungi have been extensively studied in terms of the alteration and overexpression of target proteins and the efflux and degradation of fungicides (3, 4).

Mitochondrial respiration, or oxidative phosphorylation, is a crucial cellular process of eukaryotic cells. SDHI and QoI fungicides have been developed to inhibit mitochondrial respiration, particularly the electron transport chain (ETC). These fungicides are the dominant chemicals for crop protection against fungal pathogens because of their broad antifungal spectrum against ascomycete, basidiomycete, and oomycete pathogens (5). However, as a consequence of extensive and frequent use, it has been reported that the efficacy of these fungicides is gradually declining for crop protection because of increased resistance (4). Nevertheless, because mitochondrial respiration is indispensable for various cellular functions, including energy metabolism, and respiration inhibitors exhibit a broad antifungal spectrum, mitochondrial respiration is still an attractive target for developing fungicides (6, 7). For the determination of respiration inhibition activity, many researchers have used a simple assay that compares yeast growth in two different liquid media containing a fermentable and non-fermentable carbon source as the sole carbon source (8). QoI fungicides strongly affect the growth inhibition of yeast grown in a medium containing a non-fermentable carbon source compared to growth in a fermentable carbon source (8). Therefore, this assay can discover useful natural resources, such as microbes and plant extracts, showing respiratory inhibition activity (9).

To counteract the escalating risks (e.g., toxicity and resistance issues) of chemical fungicides, interest in biological control agents (BCAs) for plant disease control has significantly increased. To find valuable BCAs, most research has focused on the antibiosis of beneficial microbes during initial screening procedures (10). The microbial strains from the genera *Bacillus*, *Pseudomonas*, and *Agrobacterium* have the most crucial role in biological control and have been commercialized as a biopesticides (11, 12). It has also been reported that several *Brevibacillus* spp. exhibit a strong antimicrobial activity against plant pathogens, indicating an excellent potential for biocontrol (13). The genus *Brevibacillus*, one of the most common genera of Gram-positive bacteria, is distributed widely in environmental habitats and includes 20 species that produce various antimicrobial peptides (AMPs), such as tostadin, gramicidins, edeines, and tyrocidines (14). AMPs made by *Brevibacillus* spp. are structurally diverse and can be classified based on their biosynthesis pathway and structural traits (14). Moreover, these AMPs can be recognized into two groups based on the mechanism of antimicrobial action: (i) AMPs targeting cell-surface components such as cell wall and membrane-bound protein and (ii) AMPs targeting intracellular components such as ribosomes and the nucleic acid synthesis machinery (14). Although the antimicrobial mechanisms of AMPs have been studied extensively, more information is still needed about the detailed mode of action in fungal organisms, particularly in plant pathogenic fungi.

As a necrotrophic fungus, *Fusarium graminearum* is a highly destructive pathogen causing severe diseases in cereal crops, such as maize, wheat, barley, and rice (15). The infection of this pathogen causes yield loss of crops but also contaminates grains with mycotoxins that are harmful to humans and animals (15). Control of Fusarium head blight (FHB) caused by *F. graminearum* is highly dependent upon the application of synthetic fungicides; however, many researchers are greatly concerned about these chemical fungicides, such as carbendazim, tebuconazole, and azoxystrobin that trigger mycotoxin deoxynivalenol (DON) biosynthesis (16, 17). Biocontrol with BCAs can be an alternative approach to chemical control and could be used as part of the integrated management of FHB and mycotoxin production (18). This study aimed to find a useful microbe exhibiting respiratory inhibitory activity, considering that SDHI and QoI fungicides targeting mitochondrial respiration are biologically active against all the stages of fungal growth (5, 19). Furthermore, an initial discovery that the culture filtrate (CF) of the *Brevibacillus brevis* HK544 strain inhibited mitochondrial respiration led to the discovery of an active compound edeine B_1_ (EB_1_) responsible for the respiration inhibition activity. The biological function of EB_1_ through transcriptome analysis, drug-induced haploinsufficiency (DIH) analysis, and targeted mutagenesis revealed that EB_1_ has effects on multiple aspects of respiration and that most of the mitochondrial-related genes in *F. graminearum* are downregulated by EB_1_. With an emphasis on understanding the molecular basis of EB_1_ in antifungal activity, our findings show that the *B. brevis* HK544 strain could be a useful resource for developing new natural agents to control FHB.

## RESULTS

### *B. brevis* HK544 exhibited a respiration inhibitory activity with a robust antifungal activity

**Fig 1:**
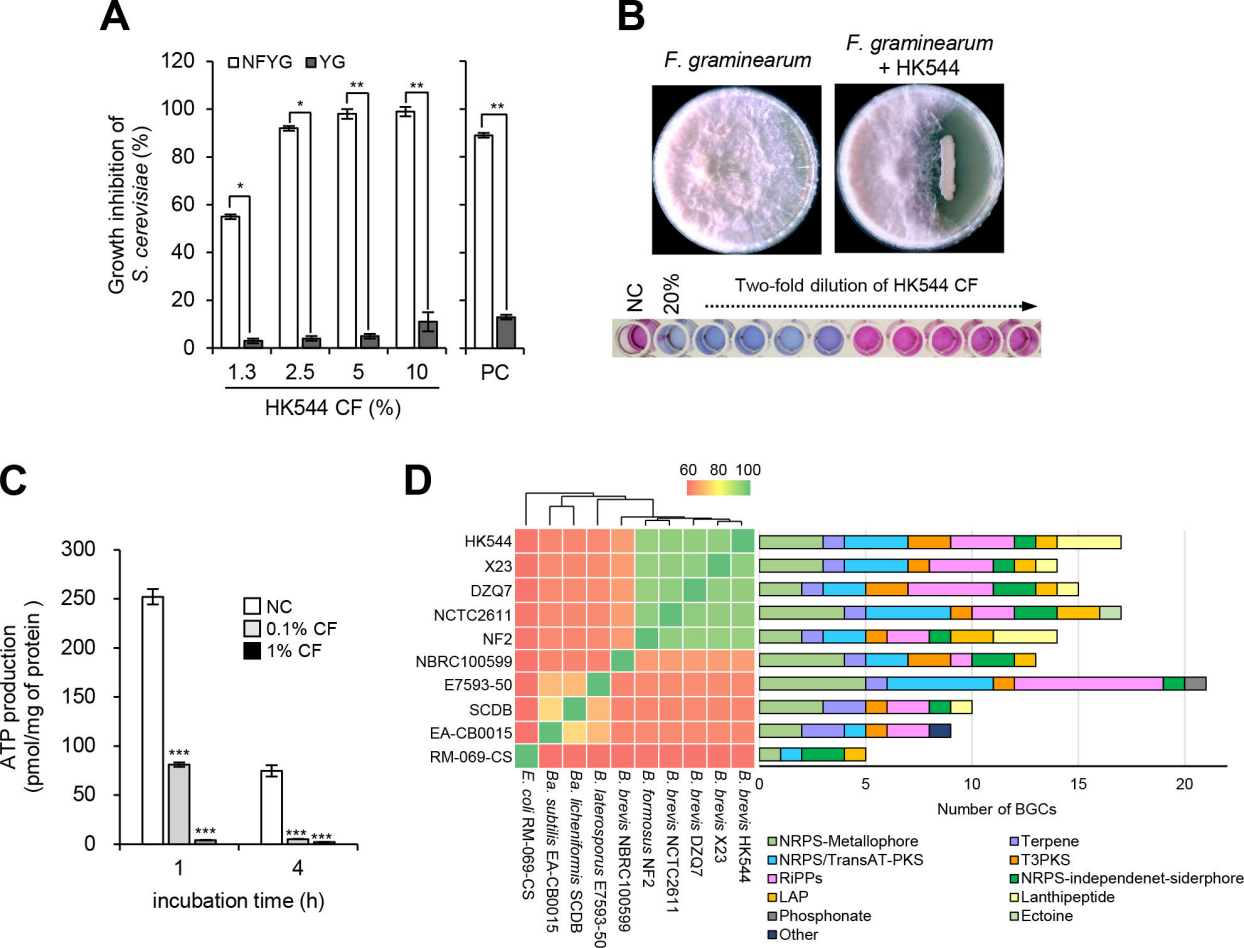
Discovery of *B. brevis* HK544 exhibiting a respiratory inhibitory activity. (**A**) Growth comparison of *S. cerevisiae* in NFYG and YG media containing HK544 CF. PC, treatment of kresoxim-methyl (10 μg/mL). (**B**) Antifungal activity of *B. brevis* HK544. The upper layer shows a dual culture assay of HK544 strain against *F. graminearum* grown on PDA+TSA (1:1, vol/vol) medium, and the photos were taken 4 days post-inoculation (dpi) at 25°C. The bottom layer shows the antifungal activity of the HK544 CF against an *F. graminearum* conidial suspension. After a 24-h incubation, each well was stained with PrestoBlue reagent. (**C**) ATP production of *F. graminearum* mycelia treated with HK544 CF. (**D**) Genome-wide comparative analysis. Heatmap and dendrogram of average nucleotide identity based on the whole genome illustrating the phylogenetic relationships between the HK544 strain and nine other bacterial strains. Bars depict the number of biosynthetic gene clusters (BGCs) belonging to each genome, which were identified by antiSMASH analysis. Colored boxes represent different BGC types as provided by the legend. Asterisks indicate a statistically significant difference in mean values (*n* = 3; *P < 0.05; **P < 0.01; ***P < 0.001). NC, negative control.

In the search for new natural resources exhibiting respiratory inhibition activity, we found that the HK544 CF (20%, vol/vol) inhibited the growth of *Saccharomyces cerevisiae* in the NFYG medium but not in the YG medium, which was similar to the strobilurin fungicide kresoxim-methyl that has a role as a mitochondrial cytochrome bc1 complex inhibitor (see Fig. S1). At various concentrations, HK544 CF inhibited the growth of *S. cerevisiae* in the range of 55%–99% when they were grown in NFYG medium, whereas the growth inhibition of *S. cerevisiae* grown in YG medium ranged from 3% to 11% (Fig. 1A). These results suggest that the HK544 strain might produce active compounds exhibiting a respiratory inhibition activity against *S. cerevisiae*.

To explore the HK544 strain in the control of plant pathogenic fungi, we investigated the antifungal activity of HK544 against *F. graminearum* as a model pathogenic fungus in terms of mycelial and conidial growth. As a result, we observed that the HK544 strain inhibited the mycelial growth of *F. graminearum* based on the dual culture assay. The HK544 CF also had a minimum inhibitory concentration (MIC) of 1.25% against *F. graminearum* when a conidial suspension was used (Fig. 1B). To investigate whether the HK544 CF has a respiratory inhibition activity against *F. graminearum*, we measured the ATP production of *F. graminearum* with and without HK544 CF treatment. When the mycelia were treated by 0.1% and 1% of the HK544 CF, the ATP production decreased by 21.9% and 64.5% compared to the non-treatment control after a 1 h treatment incubation, respectively. At 4 h after the treatment, the ATP production decreased by 83.5% and 100% compared to the non-treatment control (Fig. 1C). These results support the possibility that the HK544 CF is involved in the respiratory inhibition of *F. graminearum*.

The HK544 strain was previously identified as a *B. brevis* at the genome level (20). Prior to the chemical identification of the respirator inhibitor from *B. brevis* HK544 CF, we analyzed and compared the secondary metabolites derived from the HK544 genome with several *Brevibacillus* and *Bacillus* species (21, 22). Based on the antiSMASH analysis, we found that *B. brevis* HK544 contains 17 biosynthesis gene clusters (BGCs), including non-ribosomal peptide, polyketide synthases, and post-translationally modified peptide clusters, suggesting that the HK544 strain can produce various bioactive compounds such as tyrocidine and zwittermicin A (Fig. 1D). When compared with other *Brevibacillus* spp., the composition of HK544 BGCs was similar with other *Brevibacillus* spp. although the number of BGCs differed (Fig. 1D). Based on the previously reported bioactive compounds from *B. brevis*, limited information is available regarding the respiratory inhibitory activity of the bioactive compounds by *B. brevis*.

### *B. brevis* HK544 produced EB_1_ as a major active compound for respiratory inhibition

**Fig 2:**
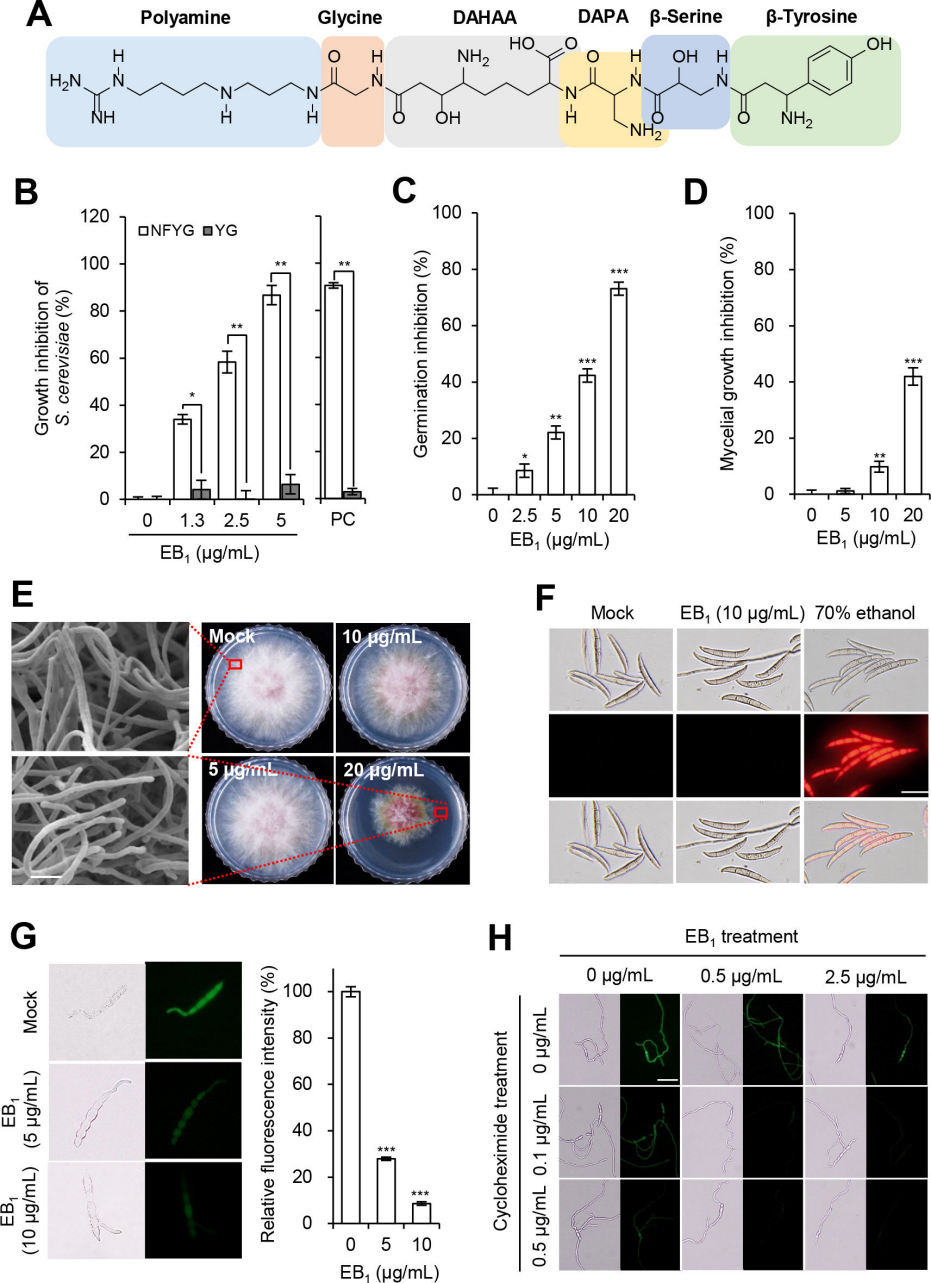
Identification of EB_1_ from *B. brevis* HK544 as an active compound for respiratory inhibition. (**A**) Chemical structure of EB_1_. DAPA, α,β-diamino-propionic acid; DAHAA, α-2,6-diamino-7-hydroxyazelaic acid. (**B**) Respiratory inhibition activity of EB_1_ based on the growth comparison of *S. cerevisiae* in NFYG and YG media containing EB_1_. PC, treatment of kresoxim-methyl (10 μg/mL). (**C**) Inhibitory effects of EB_1_ on the conidial germination of *F. graminearum*. (**D**) Inhibitory effects of EB_1_ on the mycelial growth of *F. graminearum*. (**E**) Mycelial growth and morphological observation of *F. graminearum* on complete medium (CM) supplemented with EB_1_. Scale bar, 10 μm. (**F**) Effects of EB_1_ on fungal membrane permeability. The conidial suspension was treated with EB_1_ and then stained with propidium iodide. The conidia were treated with 70% aqueous ethanol as a positive control. Scale bar, 50 µm. (**G**) Inhibition of protein synthesis by EB_1_ in germinating conidia of *F. graminearum* wild-type strain. Newly synthesized proteins were visualized using the Click-iT protein synthesis assay kit and quantified by relative fluorescence intensity. Scale bar, 50 µm. (**H**) Inhibitory effects of the combinations of EB_1_ and cycloheximide on protein synthesis of *F. graminearum* wild-type strain. Scale bar, 50 µm. Asterisks indicate a statistically significant difference in mean values (*n* = 3; *P < 0.05; **P < 0.01; **P < 0.001).

To isolate the active compounds showing respiratory inhibition activity from the *B. brevis* HK544 CF, we performed chromatography analyses based on the growth comparison of *S. cerevisiae* in NFYG and YG media. Consequently, the active compound HKC1 (117 mg) was obtained as colorless needles from the HK544 CF. The UV/Vis spectrum of HKC1 showed an end absorbance below 210 nm. The electrospray ionization mass spectrometry (ESI-MS) spectrum of HKC1 showed molecular ions at *m*/*z* 797 [M+H]^+^ and m/z 819 [M+Na]^+^. The nuclear magnetic resonance (NMR) data of HKC1 (see Table S1) wholly agreed with those of EB_1_, which is comprised of β-tyrosine, β*-*serine, α*,*β-diamino-propionic acid, α-2,6-diamino-7-hydroxyazelaic acid, glycine, and polyamine (Fig. 2A) (23). EB_1_ inhibited the growth of *S. cerevisiae* in the NFYG medium in a dose-dependent manner. In contrast, growth inhibition in the YG medium was less than 6% at the tested concentrations (Fig. 2B). These results suggest that EB_1_ has effects on the respiration of *S. cerevisiae*.

To explore how EB_1_ inhibits the growth of *F. graminearum*, we investigated the inhibitory activity of EB_1_ for conidial germination and mycelial growth. When *F. graminearum* conidia were treated with EB_1_, conidial germination was inhibited in a dose-dependent manner compared to the non-treatment control; at a concentration of 20 µg/mL, the conidial germination was inhibited by 75% (Fig. 2C). Furthermore, at the same concentration, we observed that EB_1_ has higher antifungal activity for inhibiting conidial germination compared to mycelial growth (Fig. 2C and D). Considering that AMPs can cause morphological changes in the plasma membrane and cell wall and increase cell permeability, we observed morphological alterations on *F. graminearum* grown on potato dextrose agar (PDA) medium supplemented with EB_1_. The morphology of hyphae treated with EB_1_ was similar to that of the normal control hyphae based on SEM observation (Fig. 2E). When conidia treated with EB_1_ (10 µg/mL) were stained by propidium iodide, the EB_1_-treated conidia exhibited no red fluorescence similar to the non-treatment control (Fig. 2F). By contrast, ethanol-treated conidia as a positive control exhibited a much stronger red fluorescence than that of the control and EB_1_ treatment (Fig. 2F), suggesting that EB_1_ did not trigger fungal cell membrane defects. Given that EB_1_ exhibits respiratory inhibition activity, we observed the mitochondria of *F. graminearum* treated with EB_1_ using transmission electron microscopy. Our results showed that there were no observable morphological changes in mitochondrial structure by EB_1_ treatment (see Fig. S2). Furthermore, along with no morphological changes of mitochondria, we observed there was no change in mitochondrial superoxide generation in *F. graminearum* treated with EB_1_ (see Fig. S3). Taken together, our results suggest that EB_1_ seems to indirectly regulate mitochondrial respiration in *F. graminearum*.

Considering that the peptide antibiotic edeine binds between the P-site and the E-site of the small subunit of the ribosome and consequently impairs protein synthesis (24), we investigated the effects of EB_1_ on protein synthesis based on fluorescence in fungal cells. As a result, we observed strong green fluorescence signals from the germinated conidia of *F. graminearum* (Fig. 2G). However, when *F. graminearum* was treated with 5 and 10 µg/mL of EB_1_, the fluorescence intensity was reduced by 72% and 91%, respectively (Fig. 2G); as a positive control, we observed that a translation inhibitor cycloheximide treatment also exhibited a reduced fluorescence intensity (Fig. 2H). Furthermore, when *F. graminearum* was co-treated with EB_1_ and cycloheximide, the fluorescence intensity decreased more than that of the single treatment, supporting that EB_1_ and cycloheximide target different sites on the eukaryotic ribosome (24).

### EB_1_ downregulated genes involved in oxidative phosphorylation

**Fig 3:**
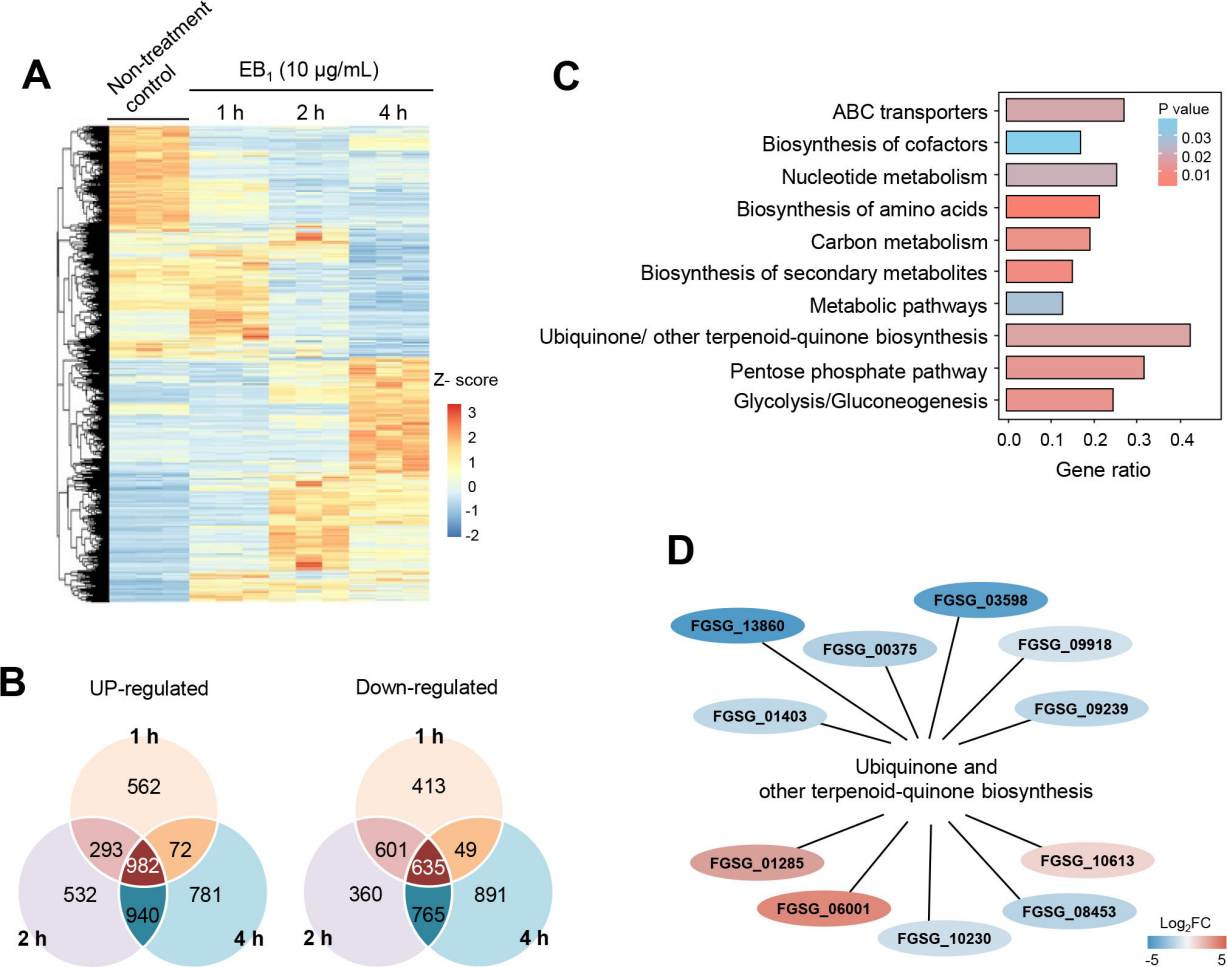
Transcriptome analysis of *F. graminearum* treated with EB_1_. (**A**) Hierarchical clustering heat map of DEGs in three biological replications of different time points after the EB_1_ treatment. The red and blue colors denote highly and weakly expressed genes, respectively. The color gradient represents the Z-score for the log_2_ FC-based normalized values. (**B**) Venn diagrams representing the number of differentially up- and downregulated genes. The number of overlapping genes is found in both conditions. (**C**) Top 10 enriched KEGG pathways of the DEGs. (**D**) Visualization of the expression of genes enriched in the KEGG pathway “ubiquinone and other terpenoid-quinone biosynthesis.” The color gradient represents the fold change value of genes from the EB_1_ 4-h treatment compared to the non-treatment control.

Considering that edeines have been widely used as transcriptional inhibitors to study ribosomal function and protein synthesis (25), we performed transcriptomic analysis of the *F. graminearum* wild-type strain from which the fungal mycelia were treated with EB_1_ and incubated for 1, 2, and 4 h (Fig. 3A). We considered differentially expressed genes (DEGs) showing differences in transcript accumulation with a log_2_ fold change of greater than 1 or less than −1 (*P* < 0.05). Compared to the non-treatment control, a total of 7,876 DEGs were identified from *F. graminearum* mycelia, with 982 and 635 genes exhibiting a significant increase and decrease in all time points, respectively (Fig. 3B). Kyoto Encyclopedia of Genes and Genomes (KEGG) enrichment analysis on the 1,617 DEGs revealed that the up- and downregulated genes were mainly enriched in “ubiquinone and other terpenoid-quinone biosynthesis (fgr00130),” “pentose phosphate pathway (fgr00030),” “glycolysis/gluconeogenesis (fgr00010),” “ABC transporters (fgr02010),” and “nucleotide metabolism (fgr01232)” (Fig. 3C). In particular, all analyzed genes within the “ubiquinone and other terpenoid-quinone biosynthesis” pathway exhibited changes in expression levels in the samples treated with EB_1_ for 4 h (Fig. 3D). Given the crucial role of ubiquinone as a key component of the electron transport chain in the oxidative phosphorylation process (26, 27), these results imply that EB_1_ has effects on oxidative phosphorylation. Furthermore, when Gene Ontology (GO) enrichment analysis was performed, the DEGs were highly enriched in GO terms related to ribosomal function such as rRNA processing (GO:0006364), ribosome biogenesis (GO:0042254), and ribosomal large subunit assembly (GO:0000027) in the biological process category (see Fig. S4). In the category of molecular function, ATP binding (GO:0005524) was a dominant term, and translation initiation-related GO terms were identified, including translation initiation factor activity (GO:0003743), RNA helicase activity (GO:0003724), and translation initiation factor binding (GO:0031369). For the cellular component, the DEGs were enriched in nucleolus (GO:0005730), mitochondrion (GO:0005739), and mitochondrial inner membrane (GO:0005743). These results suggest that EB_1_ may play an important role in the ribosome biogenesis, translation, and mitochondrial function of *F. graminearum*.

**Fig 4:**
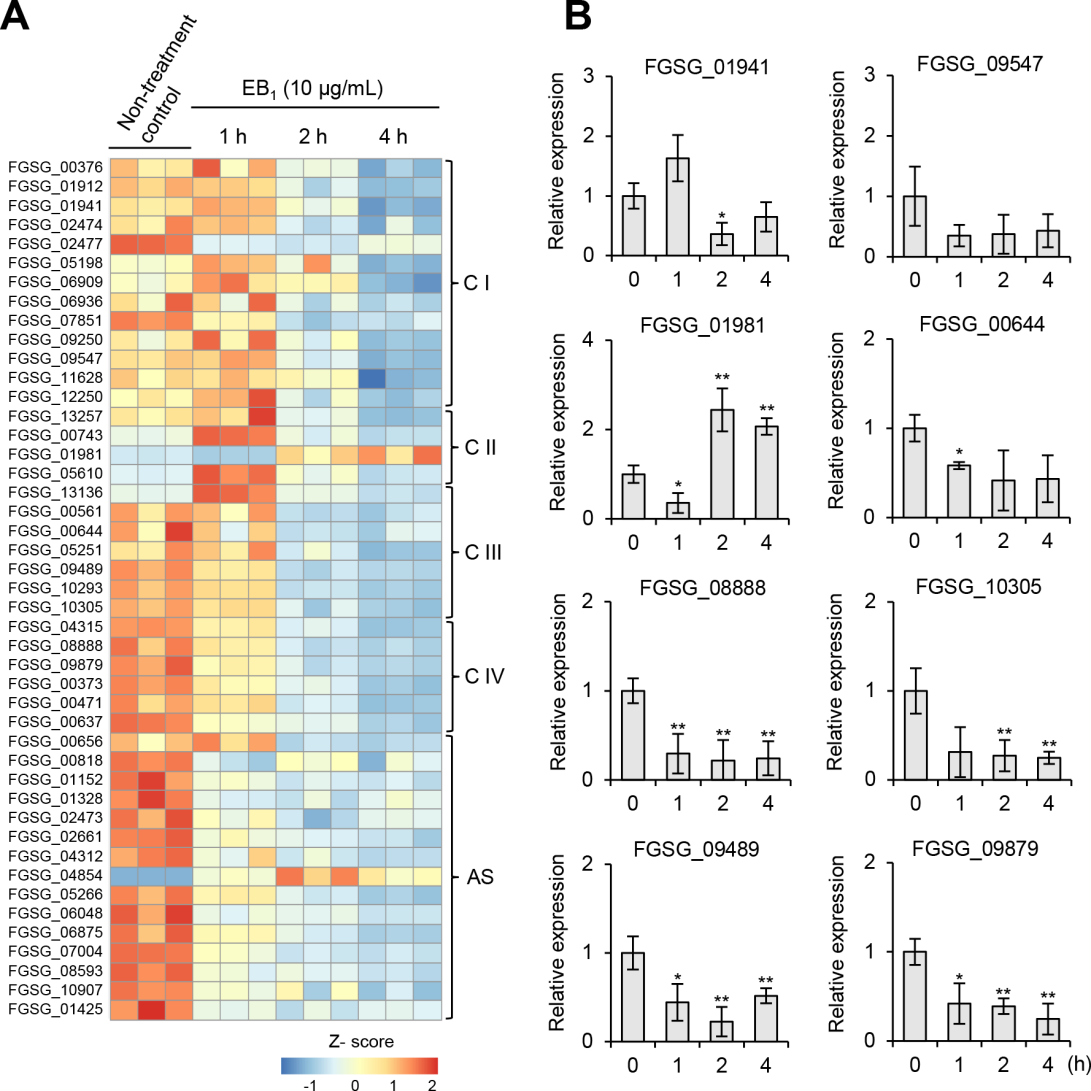
Differential expression of genes involved in oxidative phosphorylation of *F. graminearum*. (**A**) Heatmap visualization of transcriptional profiles of oxidative phosphorylation genes. The red and blue colors denote highly and weakly expressed genes, respectively. The color gradient represents the Z-score for the log_2_ FC-based normalized values. C I–IV, respiratory chain complex I–IV; AS, ATP synthase. (**B**) Validation of the representative DEGs using qRT-PCR. Asterisks indicate a statistically significant difference in mean values (*n* = 3; *P < 0.05; **P < 0.01).

Considering that EB_1_ exhibited a respiratory inhibition activity against *S. cerevisiae*, we further explored genes involved in oxidative phosphorylation of *F. graminearum* based on the KEGG database. From the RNA-Seq results, we selected 47 genes for the ETC of mitochondria. These genes were mostly downregulated 2 and 4 h after EB_1_ treatment, except for FGSG_01981 and FGSG_04854, which encode succinate dehydrogenase and ATP synthase, respectively (Fig. 4A). To validate the RNA-Seq results, we arbitrarily selected eight genes, seven downregulated genes, and one upregulated gene, for quantitative real-time PCR (qRT-PCR) analysis. Although the fold changes were different, the expression trends of these eight genes were consistent in both the RNA-Seq and qRT-PCR results (Fig. 4B).

### Haploinsufficiency profiling of *Schizosaccharomyces pombe* strains treated with EB_1_

**Fig 5:**
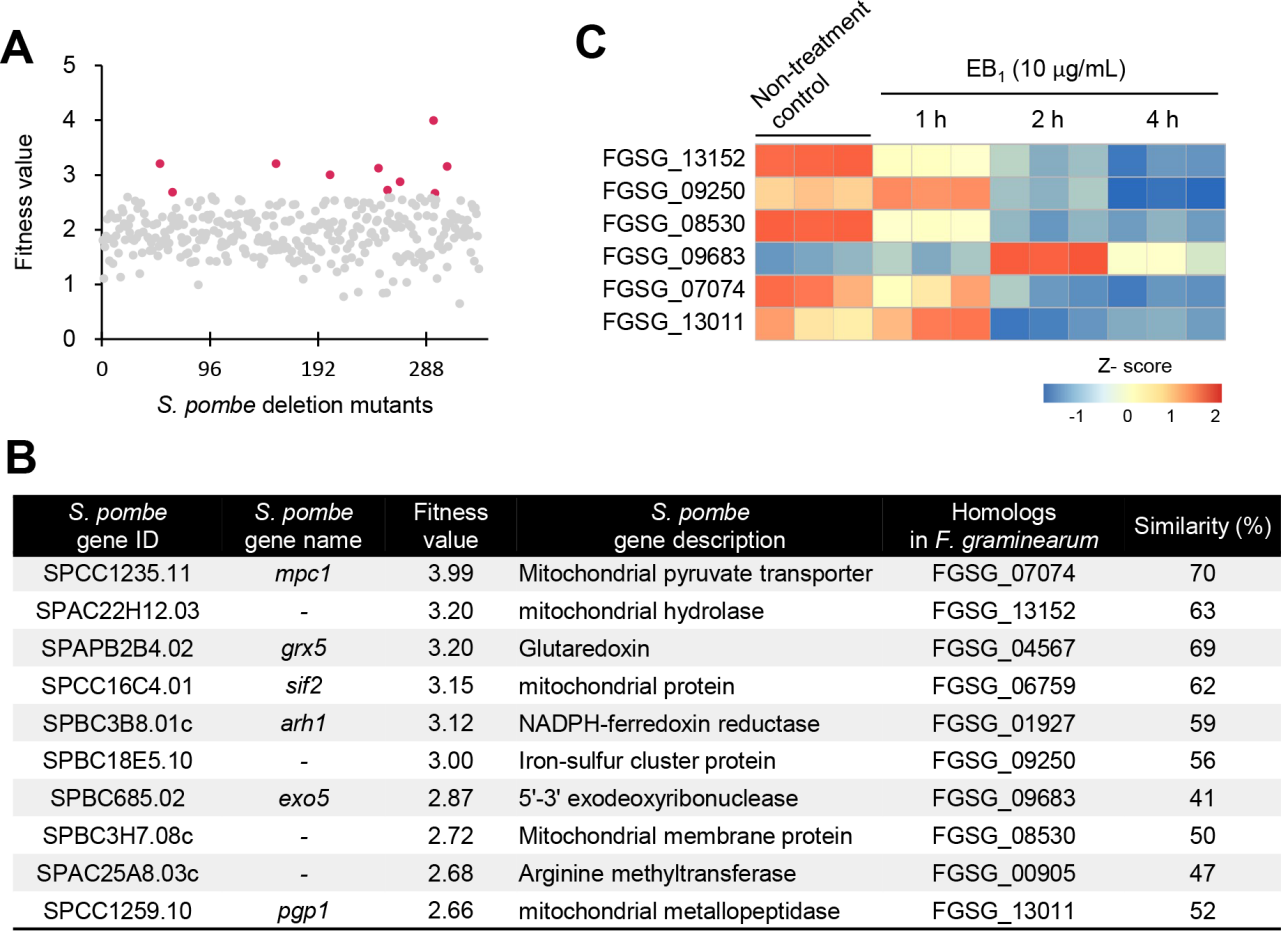
Identification of potential targets of EB_1_ using the *S. pombe* mutants. (**A**) *S. pombe* deletion mutants of the mitochondrial-related functional group subset were treated with the GI_50_ value (80 μM) of EB_1_. The fitness values of each mutant based on the cell growth inhibition were plotted as the mean of three biological replicates. Red dots indicate the top 10 target candidates of EB_1_. (**B**) Description of the top 10 target candidates of EB_1_. (**C**) Transcript levels of *F. graminearum* homologs to the target candidates derived from the RNA-Seq results. The red and blue colors denote highly and weakly expressed genes, respectively. The color gradient represents the Z-score for the log_2_ FC-based normalized values.

To further investigate mitochondrial-related genes affected by EB_1_, we exploited a DIH assay with heterozygous deletion mutants of 342 genes related to mitochondria among a *Schizosaccharomyces pombe* genome-wide deletion mutant library (4,845 genes). EB_1_ showed potent antifungal activity against wild-type *S. pombe* cells for 21 h (GI_50_ = 80 µM; see Fig. S5). DIH by EB_1_ in various *S. pombe* heterozygous deletion mutants revealed that the fitness scores of 10 gene deletion mutants were over 2.5 among the mutants, and a *mpc1*-deleted strain had the highest fitness value of 3.99 (Fig. 5A and B). Among the *F. graminearum* homologs to the 10 *S. pombe* genes, we found that six homologs were differentially expressed in response to EB_1_ (Fig. 5C). In particular, two genes FGSG_09250 encoding NADH dehydrogenase flavoprotein 1 in mitochondrial respiratory chain complex I and FGSG_07074 encoding mitochondrial pyruvate carrier 1 were assigned to the oxidative phosphorylation pathway (fgr00190) and mitochondrial biogenesis (fgr03029) of *F. graminearum* KEGG database, respectively. To further determine whether the target deletion of six homologs (FGSG_07074, 08530, 09250, 09683, 13011, and 13152) leads to chemical sensitivity to EB_1_, we generated gene deletion strains (see Fig. S6) and compared the growth of the wild-type and the deletion strains according to the treatment of EB_1_. As a result, we found that there was no significant difference in EB_1_ sensitivity between the wild-type and deletion strains (see Fig. S7).

### Deletion of *FgSdhC1* increased the sensitivity to EB_1_

**Fig 6:**
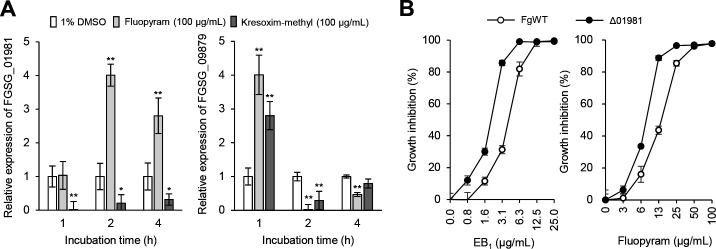
Effects of chemical fungicides and EB_1_ on *F. graminearum* strains. (**A**) Transcript levels of the genes encoding the putative succinate dehydrogenase (FGSG_01981) and cytochrome c oxidase (FGSG_09879) subunit by treatment of chemical fungicides. (**B**) Growth inhibition effects of EB_1_ and fluopyram on the Δ01981 strain. FgWT, *F. graminearum* wild-type strain; Δ01981, an FGSG_01981 gene deletion strain. Asterisks indicate a statistically significant difference in mean values (*n* = 3; *P < 0.05; **P < 0.01).

Considering that EB_1_ changed the expression of genes involved in oxidative phosphorylation, we explored whether SDHI and QoI fungicides have effects on the transcript level of succinate dehydrogenase (*FgSdhC1*; FGSG_01981) and cytochrome c oxidase (*FgCox7A*; FGSG_09879) subunits. They were selected because their expressions were significantly increased and decreased by EB_1_, respectively (Fig. 6A). *FgSdhC1* was highly induced and suppressed by the SDHI fungicide fluopyram and QoI fungicide kresoxim-methyl, respectively, and both chemicals suppressed *FgCox7A*. These results were sillier than the RNA-Seq and qRT-PCR results derived from the EB_1_ treatment. However, in contrast to the EB_1_ treatment results, we observed that *FgCox7A* was induced by both chemicals 1 h after the treatment (Fig. 6A).

To further determine whether the deletion of succinate dehydrogenase genes leads to chemical sensitivity to EB_1_, we generated an *FgSdhC1* deletion strain that was named Δ01981 (see Fig. S8) and compared the growth of the wild-type and Δ01981 strains according to the treatment of EB_1_. As a result, the Δ01981 strain was more sensitive to EB_1_ compared to the wild-type strain below a concentration of 12.5 µg/mL; in particular, the greatest difference (2.7-fold) was shown at a concentration of 3.1 µg/mL (Fig. 6B). In addition, when we investigated the growth difference to fluopyram which binds to the ubiquinone-binding sites in the succinate dehydrogenase complex composed of four subunits (SdhA, SdhB, SdhC, and SdhD), the deletion of *FgSdhC1* also led to a change in fluopyram sensitivity (Fig. 6B). Therefore, our observations that the deletion of *FgSdhC1* increased the sensitivity to EB_1_ support that EB_1_ has effects on the mitochondrial respiration of *F. graminearum*.

### HK544 CF and EB_1_ reduced the development of FHB

**Fig 7:**
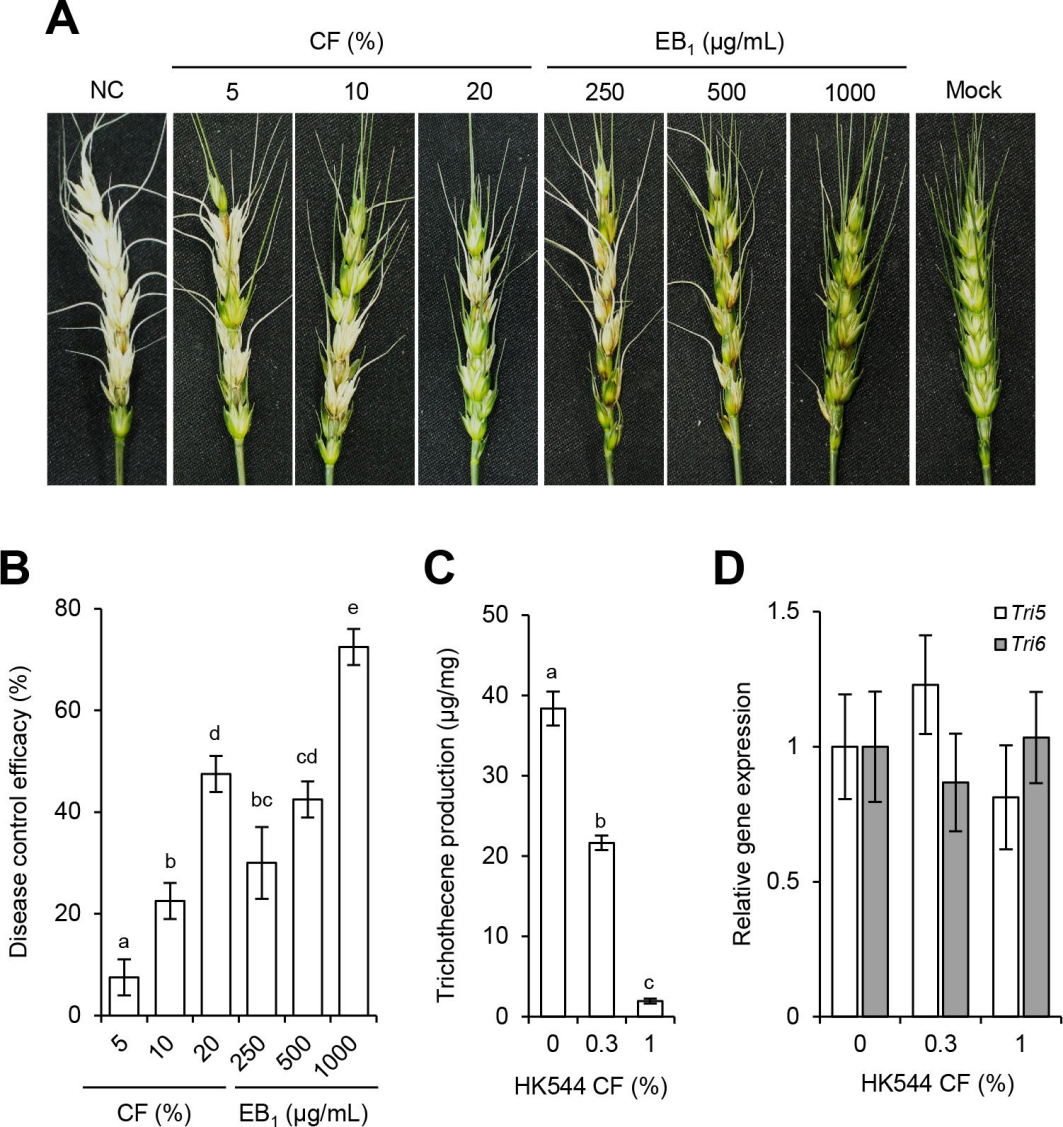
FHB control efficacy and trichothecene analysis. (**A**) Each wheat head was inoculated with an *F. graminearum* conidial suspension (1 × 10^6^ conidia/mL) 24 h after treatment with HK544 CF and EB_1_. NC, non-treatment control; Mock, mock inoculation was performed with the 0.025% Tween 20 solution. The photos were taken at 5 dpi. (**B**) The disease control efficacy (%) was calculated by the lesion area (%) of wheat heads compared to the non-treatment control. (**C**) Trichothecenes were extracted from 7-day-old cultures in liquid minimal medium including agmatine (MMA) supplemented with or without HK544 CF and quantified by gas chromatograph-mass spectrometer (GC-MS). (**D**) Transcript levels of the trichothecene biosynthetic genes *Tri5* and *Tri6* were measured by qRT-PCR from 4-day-old cultures in liquid MMA medium supplemented with or without HK544 CF. Different letters indicate a statistically significant difference at *P* < 0.05.

As proof of concept that the *in vivo* antifungal activity of HK544 CF and EB_1_ can be used as a BCA, we investigated their ability to control or reduce the FHB by treating flowering wheat heads with HK544 CF and EB_1_ before inoculation with *F. graminearum* conidial suspension. The non-treatment control exhibited typical head blight symptoms, which manifested as discoloration, at 5 days post-inoculation (dpi), whereas the treatments with HK544 CF and EB_1_ led to suppressed FHB development in a dose-dependent manner (Fig. 7A and B). In addition, given that chemical fungicides inhibiting mitochondrial respiration induce trichothecene production of *F. graminearum* (16, 17), we investigated whether HK544 CF containing EB_1_ induces trichothecene production. The results showed that the cultures treated with HK544 CF produced reduced trichothecenes in a dose-dependent manner without the differential expression of the trichothecene biosynthetic genes *TRI5* (FGSG_03537; trichodiene synthase) and *TRI6* (FGSG_03536; transcription factor) between the treated and untreated cultures (Fig. 7C and D). This result suggests that the reduced trichothecene production is from the inhibition of fungal growth by HK544 CF. Taken together, our results indicate that *B. bacillus* HK544 CF can control FHB with a reduction of trichothecene production.

### Synergistic effects of the combinations of EB_1_ and respiratory inhibitors

**Fig 8:**
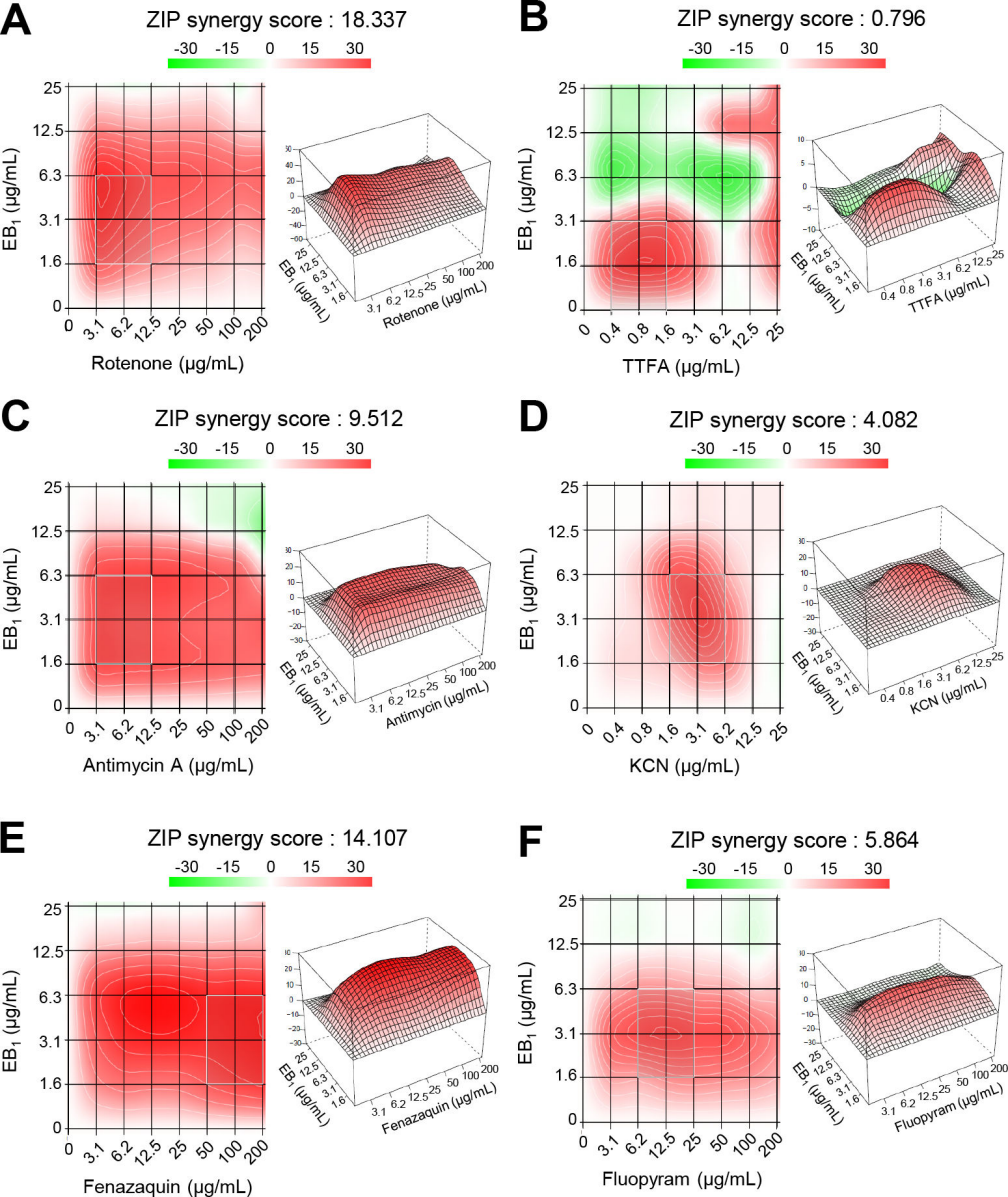
Synergistic effects of the combinations of EB_1_ and respiratory inhibitors on *F. graminearum*. In each drug combination (A–F), ZIP synergy scores based on the *F. graminearum* growth were calculated by the SynergyFinder software, and the interaction landscapes are shown in both 2D (left) and 3D (right) with red color (synergism) and green color (antagonism). The summary ZIP score calculated over the full dose-response matrix and landscape is marked on top of the panels. The gray-shaded area with a white line indicates the most synergistic area in that particular dose-response matrix.

Considering that the combination assay can provide information on improved antifungal potency for the application and also help unravel the mechanism of action of the drugs, we investigated the synergistic effects between EB_1_ and ETC inhibitors (rotenone for complex I, thenoyltrifluoroacetone (TTFA) for complex II, antimycin A for complex III, and potassium cyanide (KCN) for complex IV) against *F. graminearum*. First, we determined the MIC values of each ETC inhibitor against *F. graminearum*. The results showed that the MICs of rotenone and antimycin A were above the highest concentration tested (>200 µg/mL), and TTFA and KCN had MIC values of 25 and 12.5 µg/mL, respectively (Fig. 8). Because the fractional inhibitory concentration index estimates the drug interactions at the MIC levels, we used a zero interaction potency (ZIP) model to understand the interactions over the entire range of concentrations (28). When EB_1_ (ranging from 1.6 to 25  µg/mL; MIC value of 12.5  µg/mL; see Fig. S9) was combined with ETC inhibitors, the highest average synergy score (18.337) was observed in the combination of EB_1_ + rotenone among the tested combinations although the 2D and 3D plots of the model-specific statistic showed regions of synergy in all the tested combinations (Fig. 8). An EB_1_ + TTFA combination exhibited the lowest average synergy score (0.796) and also showed regions of antagonism in the matrix (Fig. 8).

Based on our observation that the combinations EB_1_ + rotenone and EB_1_ + TTFA exhibit the highest and lowest average synergy scores, respectively, we further investigated the synergistic interactions between EB_1_ and commercial fungicides targeting complexes I and II. When EB_1_ was combined with the complex I inhibitor fenazaquin (MIC, >200 µg/mL) and complex II inhibitor fluopyram (MIC, 100 µg/mL), the average ZIP score was 14.107 and 5.864 (Fig. 8), respectively, suggesting that there was a synergistic effect between EB_1_ and the complex I inhibitor fenazaquin. These results show that EB_1_ synergistically interacts with complex I inhibitors rotenone and fenazaquin for the respiratory inhibition of *F. graminearum*.

## DISCUSSION

The mitochondrial machinery facilitating ATP synthesis by oxidative phosphorylation is a promising target for active compounds derived from microbial metabolites (9, 29). The natural compound strobilurin A was discovered in the wood-rotting basidiomycete fungus *Strobilurus tenacellus*, and synthetic strobilurin fungicides (e.g., azoxystrobin and kresoxim-methyl) were developed from β-methoxyacrylate through optimization of their photostability and activity (5). Although fungicides targeting mitochondrial respiration are the dominant chemical groups used in the market (30), it has been reported that strobilurin fungicides do not inhibit the respiration of several fungal pathogens, such as *Magnaporthe oryzae* and *Botrytis cinerea*, in which an alternative oxidase is activated (31–33). Respiration is maintained through an alternative pathway when strobilurin binds to cytochrome bc_1_ complex in the ETC (31–33). Due to issues of resistance and reduced effectiveness for these chemicals in several fungal pathogens, this study aimed to find natural resources exhibiting respiratory inhibitory activity. To this end, numerous microbial CFs were explored using a yeast screening system for their respiratory inhibitory activity. As a result, we found a soil-borne microbe *B. brevis* HK544 strain, and identified EB_1_ as an active compound for the respiration inhibition activity.

*Brevibacillus* is a genus of bacteria reclassified from *Bacillus* based on the 16S rRNA sequence analysis (14). This genus has been considered a rich resource for antibacterial and antifungal activity with the isolation and characterization of many active compounds such as gramicidin S, tyrocidines, tauramamide, bogorols, and laterosporulin (14, 34–36). Most of the peptides produced by *Brevibacillus* have been known to damage the cytoplasmic membrane, but edeines exhibit a different mode of action from most *Brevibacillus* peptides (14). Edeines initially identified in *B. brevis* Vm4 are linear non-ribosomal peptides with a structurally unusual backbone that includes four non-proteinogenic amino acids and an N-terminal polyamine cap (37). This unique structure of edeines contributes to various biological functions, inhibiting the growth of numerous bacteria, fungi, mycoplasma, and tumor cells (38, 39). Furthermore, it has been reported that low concentrations of edeines inhibit DNA synthesis (22, 40). In contrast, high concentrations of edeine prevent translation initiation by inhibiting the binding of fMet-tRNA to the P site of the 30S ribosomes in prokaryotes (22, 41). Although significant progress has been made by the docking model between edeine and prokaryotic/eukaryotic ribosomes, relatively little is known mechanistically about the biological function of edeines, particularly against plant pathogenic fungi. In this study, EB_1_ exhibited a respiratory inhibition activity against the necrotrophic fungus *F. graminearum* with low ATP production and downregulation of ETC genes. Additionally, the deletion of *FgSdhC1*, one of the succinate dehydrogenase complex components in ETC, increased the sensitivity of *F. graminearum* to EB_1_. With previous reports that edeines inhibit protein synthesis by binding to ribosomes, our results postulated that EB_1_ inhibits the growth of *F. graminearum* by inhibiting mitochondrial translation. This hypothesis can be explained by the fact that some ribosome-targeting antibiotics can inhibit mitoribosomes, considering that mitochondria are of prokaryotic origin, and the bacterial and mitochondrial protein translation machinery share similarities (42, 43). Indeed, tetracycline binding to ribosome subunits has been reported to interfere with mitochondrial proteostasis, leading to changes in nuclear gene expression and altered mitochondrial dynamics and function in eukaryotes (44). When we calculated the predicted binding free energies with molecular dynamics simulations to compare the binding affinity of EB_1_ to mitochondrial ribosome and cytosolic ribosome, our results showed that the EB_1_ in the mitochondria ribosome exhibited higher binding affinity than cytosolic ribosome although the EB_1_ can bind to both ribosomes which were located in cytosolic and mitochondria (see Table S2). Therefore, our results cannot exclude the possibility that inhibition of mitochondrial respiration by EB_1_ is a major byproduct of an antibiotic-target interaction.

Biological control by antagonistic microbes, including yeasts, fungi, and bacteria, can be used to manage fungal diseases, and the BCAs can also solve the resistance and toxicity issues derived from chemical pesticides (10, 45, 46). Although numerous antagonistic strains of *Brevibacillus* spp. have been identified, most research has focused on *in vitro* antifungal activity with identifying active compounds, not disease control efficacy. In this study, we showed that the reduction of FHB by HK544 CF and EB_1_ ranged from 54% to 96% at the tested concentrations. Unlike the chemical fungicides triggering mycotoxins (16, 17), the HK544 CF reduced trichothecene production in the cultures, suggesting that *B. brevis* HK544 can decrease both fungal growth and trichothecene production. Furthermore, our observation that EB_1_ inhibited the fungal growth more effectively with the complex I inhibitor fenazaquin suggests that EB_1_ is a potent chemosensitizer. These findings provide further information for the effectiveness of its *in vivo* antifungal activity, given that the disease control efficacy of *B. brevis* HK544 seems to be insufficient when used alone. Considering that antifungal chemosensitization is a novel antifungal intervention strategy in which one drug is used to enhance the efficacy of conventional agents against fungal pathogens (46), our results suggest that *B. brevis* HK544 could be used in combination with chemical fungicides to enhance the efficacy of antifungal agents. Further research, such as the formulation of HK544, field trials with a mixture of HK544 and chemicals, and HK544 sensitivity analyses against chemical fungicides, will be necessary to prove the feasibility of the *B. brevis* HK544 as a chemosensitizer for FHB control.

Regarding *in vitro* antifungal activity, we observed that EB_1_ is more effective against conidial germination than mycelial growth. Similarly, it has been reported that chemical fungicides targeting mitochondrial respiration exhibit better fungicidal activity against spore germination than mycelial growth in many filamentous fungi (16). For example, the mycelial growth of *B. cinerea* was less sensitive to the SDHI fungicides fluopyram and boscalid compared to conidial germination (47, 48). A similar dominant effect on the inhibition of spore germination has also been described from the QoI fungicides (16). This phenomenon can be explained by the differences in the target site sensitivity and physiological processes between spores and mycelia (49). Considering that SDHI and QoI fungicides target energy production in the mitochondria, and fungal spores have a lower metabolic activity with the transient softer cell walls compared to the growing mycelia, mitochondrial respiration inhibitors can lead to the different susceptibility between spores and mycelia (5). Therefore, we speculate that EB_1_ has a similar action with SDHI and QoI fungicides in inhibiting fungal development.

In summary, our findings shed light on the *in vitro* antifungal activity of *B. brevis* HK544 and its major active compound EB_1_, with multifaceted evidence of respiratory inhibition. The promising results of the disease control efficacy in wheat heads infected with *F. graminearum* with the treatment of HK544 CF and EB_1_ highlight their practical potential in crop protection against pathogenic fungi. Furthermore, the synergistic interactions between EB_1_ and respiratory inhibitors, particularly complex I inhibitors, reveal novel opportunities for enhancing antifungal activity. Further research into the optimization of formulations and investigation of the activity spectrum could pave the way for the practical applications of HK544 containing EB_1_ as an effective biofungicide in agriculture.

## MATERIALS AND METHODS

### Microbial strains and culture conditions

The soil-borne bacterium *B. brevis* HK544 strain was isolated from the soil at Daejeon, South Korea, and deposited as a patent microorganism to the Korean Agricultural Culture Collection (KACC, Jeonju, South Korea; No. KACC 81093BP). The bacterium was maintained on tryptic soy broth (TSB; BD Difco, San Jose, CA, USA) agar plate at 30°C. For the *in vitro* and *in vivo* antifungal activity, the *F. graminearum* wild-type strain Z-3639 was used as a target fungal pathogen and grown in complete medium (CM) or potato dextrose broth (PDB; BD Difco) supplemented with 1.5% agar as needed (1). Conidia from *F. graminearum* were induced in a carboxymethyl cellulose medium (50). A minimal medium including 5 mM agmatine (MMA) was used to evaluate trichothecene production (51). All microbial strains used in this study were stored in 20% glycerol at −80°C.

### Mitochondrial respiratory inhibition assay

The inhibitory activity of mitochondrial respiration was investigated using the method described by Han et al. (9). Briefly, the growth inhibition of *S. cerevisiae* A-139 treated with CF or EB_1_ was compared in two different media: YG (1% yeast extract and 2% glucose) and NFYG (1% yeast extract and 1% glycerol). After 24-h incubation, the optical density (OD_600_) of each well was measured using a microplate reader (Bio-rad, Hercules, CA, USA), and the growth inhibition (%) of the A-139 strain was calculated as follows: [1 − (OD_600_ of treatment/OD_600_ of control)] × 100. The QoI fungicide kresoxim-methyl and 1% dimethyl sulfoxide (DMSO) were used as positive and negative controls, respectively.

### Measurement of ATP production

To measure the ATP production of *F. graminearum* treated with the HK544 CF, a conidial suspension (5 × 10^7^ conidia/mL) was inoculated into 50 mL of PDB. After 24 h incubation, HK544 CF was added to the cultures at a final concentration of 0.1% and 1%, respectively. After 1- and 4-h incubation, mycelium was harvested and ground in liquid nitrogen with a pestle and mortar. ATP production in the ground mycelia was determined with the ATP Determination Kit (Invitrogen, Eugene, OR, USA) following the manufacturer’s instructions. Fungal tissue (10 mg) was reacted with 1.25 µg/mL firefly luciferase, 50 µM D-luciferin, 1 mM dithiothreitol, and 1× reaction buffer. After a 15-min incubation, luminescence was measured by the BioTek Synergy LX multimode reader (Agilent Technologies).

### Isolation and identification of active compounds from HK544 CF

The HK544 strain was inoculated into 400 mL of TSB medium in a 2 L-Erlenmeyer flask and incubated at 25°C for 3 days with shaking (150 r/min). A total of 4 L of HK544 culture broth was centrifuged at 10,000 × *g* for 30 min and passed through Whatman No.1 filter paper (Maidstone, UK). The HK544 CF was applied onto a Diaion HP-20 column (5 × 30 cm; Mitsubishi Chemical, Tokyo, Japan) with a stepwise gradient elution of 0%, 20%, 40%, 60%, 80%, and 100% aqueous acetone to give F1–F6. The 60% aqueous methanol fraction (2.0 g) exhibiting the antifungal activity was subjected to a reversed-phase flash column chromatography packed with LiChroprep RP-18 (40–63 µm; Merck, Kenilworth, NJ, USA) and eluted with 0%, 20%, 40%, 60%, 80%, and 100% aqueous methanol (containing 0.1% formic acid). An active fraction F41 (200 mg) was further purified by a Sephadex LH 20 column (16 × 100 cm; Merck). The column was eluted at a flow rate of 0.1 mL/min with deionized water. Finally, the purified compound HKC1 (117 mg) was obtained and kept at −20°C until further analysis.

The chemical structure of HKC1 was determined by spectroscopic analyses and comparisons with values in previous literature (23). The ESI-MS data were recorded on a single-quadruple mass spectrometer (Acquity QDa; Waters, Milford, MA, USA). All nuclear magnetic resonance (NMR) spectra were recorded in deuterium oxide (Cambridge Isotope Laboratories, Tewksbury, MA, USA) at 25°C on the Bruker Advance 500 MHz spectrometer (Bruker BioSpin, Rheinstetten, Germany).

### *In vitro* antifungal activity assay

For the inhibitory effect of EB_1_ on the conidial germination of *F. graminearum*, EB_1_ dissolved in DMSO was added to the microplate wells containing a conidial suspension (5 × 10^5^ conidia/mL of PDB). After a 10 h incubation at 25°C, the number of germinated conidia was counted by microscopic observation in 100 conidia. To investigate the mycelial growth inhibition of EB_1_, a mycelial disk (5 mm in diameter) of *F. graminearum* was inoculated onto CM supplemented with different concentrations of EB_1_, and then, the radial growth of *F. graminearum* was measured at 5 dpi. To determine the MIC values of CF and EB_1_ against *F. graminearum*, we used the broth microdilution method as previously described (52). Briefly, a conidial suspension (1 × 10^4^ conidia/mL of PDB) of *F. graminearum* was added to the wells of a 96-well microtiter plate, and then, EB_1_ stock solutions dissolved in DMSO were added at an initial concentration of 25 µg/mL by twofold serial dilutions. The 1% DMSO was used for a negative control. After incubation for 24 h at 25°C, the MIC was determined by visual examination and corresponded to the lowest concentration that caused complete growth inhibition.

### Microscopic observation

The *F. graminearum* mycelia grown on CM containing the HK544 CF were observed under an FEI Quanta 250 FEG scanning electron microscope (Hillsboro, OR, USA) at 10 kV. To investigate the effect of EB_1_ on *F. graminearum* membrane permeability, the conidia were incubated with EB_1_ (12.5 µg/mL) for 4 h and then stained with 2 µM propidium iodide for 1 h. As a positive control, *F. graminearum* conidia was treated with 70% aqueous ethanol. Images were captured on an Olympus BS53 microscope (Münster, Germany) with a filter set of 494 nm/515 nm (excitation/emission wavelength).

### Protein synthesis assay

To investigate the effects of EB_1_ on protein synthesis in fungal cells, we evaluated the initial protein level using a Click-iT HPG Alexa Fluor 488 Protein Synthesis Assay kit (Thermo Fisher, Massachusetts, UK) according to the manufacturer’s instructions. Briefly, a conidial suspension (1  ×  10^6^ conidia/mL of CM) of *F. graminearum* was treated with EB_1_ (or cycloheximide) with 4 h incubation. After 1 h-additional incubation with HPG for 90  min, the fungal cells were fixed using a 3.7% formaldehyde solution and permeabilized with 0.5% Triton X-100 in PBS. The Click-iT reaction buffer containing Alexa Fluor dye with the azide moiety was added to the samples and then incubated for 30 min at room temperature in the dark. After washing with the Click-iT reaction rinse buffer, the samples were stained with 1× HCS NuclearMask blue stain solution, and the newly synthesized proteins were examined by an Olympus BS53 microscope (Münster, Germany) with a consistent exposure time. For the quantification, the fluorescence signal intensity was determined using the BioTek Synergy LX multimode reader (Agilent Technologies).

### Transcriptome analysis

The *F. graminearum* Z-3639 strain was grown in 50 mL of CM at 25°C with shaking for 72 h and then incubated with EB_1_ (10 µg/mL). After 1-, 2-, and 4-h incubation, the fungal mycelia were collected and washed twice with distilled water before being ground in liquid nitrogen. Total RNA was extracted with an Easy-Spin Total RNA Extraction Kit (iNtRON Biotechnology, Seongnam, South Korea) following the manufacturer’s instructions, and RNA quality was assessed on the Agilent 2100 Bioanalyzer system (Agilent Technologies, Santa Clara, CA, USA). The total RNA was subjected to RNA-Seq library preparation using a TruSeq Stranded Total RNA Sample Prep Kit (Illumina, San Diego, CA, USA) following the manufacturer’s instructions. Each library from three biological replicates per treatment (total 12 libraries) was sequenced for 101 bp paired-end reads with an Illumina NovaSeq6000 platform (Illumina) by Macrogen in Seoul, South Korea. The resulting sequences were analyzed using the Galaxy web-based platform. Trimming of reads was conducted using Trimmomatic (Galaxy version 0.38.1) (53), and the paired-end reads of each sample were aligned to the *F. graminearum* reference genome sequence using RNA STAR (Galaxy version 2.7.10b) (54). Gene expression quantification was computed using featureCounts (Galaxy version 2.0.3) (55), and the analysis of differential gene expression was performed using the DESeq2 R package (56). Enrichment analysis was performed in DAVID Bioinformatics Resources (57).

### Quantitative real-time PCR analysis

Total RNA was extracted using an Easy-Spin Total RNA Extraction Kit (iNtRON Biotechnology), and first-strand cDNA was synthesized from the total RNA using SuperScript III First-Strand Synthesis SuperMix (Invitrogen). qRT-PCR was performed using SsoFast EvaGreen Supermix (Bio-Rad) with the CFX Real-Time PCR System (Bio-Rad). An endogenous housekeeping gene *ubh* (FGSG_01231, ubiquitin C-terminal hydrolase) of *F. graminearum* was used as an internal control for normalization (58). Relative expression levels were calculated through the 2^−ΔΔCT^ method (59). The experiments were repeated twice, with three replicates for each. Primers used in the qRT-PCR are listed in Table S3.

### Generation of knockout strain

Gene knockout constructs were prepared by double-joint PCR as previously described (60). Briefly, the fungal genomic DNA of Z-3639 was extracted from freeze-dried mycelia powder according to the Fusarium laboratory manual (1). The 5′ and 3′ flanking regions (approximately 1.5 kb) of a target gene were amplified from *F. graminearum* gDNA and then were fused into a hygromycin resistance cassette (Hyg) amplified from pGEM-T_Hyg (61). The resulting constructs were transformed into the Z-3639 protoplasts using polyethylene glycol (PEG)-mediated transformation (50). Transformants grown on CM medium containing hygromycin were subjected to PCR-based screening using specific primer pairs. Primers used in the generation of knockout strains are listed in Table S3.

### EB_1_-induced haploinsufficiency analysis

DIH assay was performed by using *S. pombe* heterozygous deletion mutants from Bioneer (Daejeon, South Korea) based on the measurement of cell growth inhibition (62). Briefly, *S. pombe* cells were grown at 30°C in a complete YES medium containing 0.5% yeast extract, 2% glucose, and various supplements as described (63). The GI_50_ value of EB_1_ was determined against the *S. pombe* wild-type strain (SP286; h+/h+, ade6-M210/ade6-M216, ura4-D18/ura4-D18, leu1-32/leu1-32). Spore suspensions (1 × 10^6^ cells/mL of YES medium) of a mitochondrial-related functional group subset, including 342 heterozygous deletion mutants of *S. pombe*, were added to the wells of a 96-well microtiter plate. Then, the EB_1_ stock solution dissolved in DMSO was added at a final concentration of 80 µM. As a control, we used a YES medium containing 0.06% DMSO. The microtiter plates were incubated at 30°C for 20 h. The fitness value that represents the growth inhibitory potency of EB_1_ at the GI_50_ dose in each deletion mutant was calculated by dividing the cell mass (*A*_600_) of the DMSO control with the one for the treated drug. Gene information on *S. pombe* was obtained from the PomBase database (64).

### FHB disease control efficacy

To investigate the disease control efficacy of HK544 CF and EB_1_, the spray inoculation method was used with wheat (*Triticum aestivum* cv. Eunpa) that was grown in a glasshouse at 25°C ± 5°C for 4 weeks (65). Briefly, mid-anthesis stage wheat heads were sprayed with HK544 CF and EB_1_ dissolved in sterile water using a hand-held atomizer; both samples contained 0.025% Tween 20 solution as a wetting agent. The sterile water alone served as a negative control. After the treated wheat plants were air dried for 24 h, 20 of each treated wheat head were sprayed with a conidial suspension (1 × 10^6^ conidia/mL) of *F. graminearum* containing 0.025% Tween 20 solution. The inoculated plants were kept in the humidified chamber (25°C; 12 h photoperiod) for 3 days and then moved to a glasshouse at 25°C ± 5°C. After 2 days of additional incubation, we recorded a percentage of the wheat head surface showing FHB symptoms. The FHB disease control efficacy was calculated with the following equation: control efficacy (%) = 100 × (1 − *B*/*A*), where *A* is the mean of the lesion area (%) on the wheat head of the control plants and *B* is the mean of lesion area (%) on the wheat head of the treated plants (65).

### Trichothecene analysis

A conidial suspension (1 × 10^6^ conidia/mL) was incubated in MMA medium supplemented with or without HK544 CF at 25°C under stationary growth conditions to measure trichothecene production. Trichothecenes were extracted from 7-day-old MMA cultures with an ethyl acetate-methanol mixture (4:1, vol/vol) as described previously (49). Briefly, the extracts were dried and derivatized with Sylon BTZ (Supelco, Bellefonte, PA, USA). Sequentially, the derivatized samples were mixed with an equal volume of n-hexane and distilled water. Then, the hexane layer was analyzed for trichothecene production using a Shimadzu QP2020 gas chromatograph-mass spectrometer (GC-MS) (Shimadzu, Kyoto, Japan). Quantification was performed by comparing the peak areas of the samples with those of external standards of DON and 15-ADON (Sigma-Aldrich, St. Louis, MO, USA), and then normalized by biomass.

### Synergetic effect assay

As previously described, a checkerboard assay was performed to determine synergism between EB_1_ and respiratory inhibition compounds (66). Briefly, EB_1_ and each inhibitor dissolved in DMSO were added using twofold serial dilutions in the x-axis and y-axis of a 96-well microtiter plate, respectively, containing an *F. graminearum* conidial suspension (1 × 10^4^ conidia/mL). Rotenone (complex I), TTFA (complex II), antimycin A (complex III), and KCN (complex IV) were used as specific inhibitors for ETC complexes, and fenazaquin and fluopyram were used as chemical fungicides targeting complex I and II, respectively. As a negative control, we used a 1% DMSO treatment. After an 18 h incubation, the OD_600_ value of each well was measured using a microplate reader (Bio-rad), and the growth inhibition (%) was calculated as follows: [1 − (OD_600_ of combined treatment/OD_600_ of negative control)] × 100. Obtained data were analyzed with SynergyFinder (version 1.6.1), and the synergy score was evaluated based on the ZIP method (67, 68). The results were interpreted by the ZIP scores as synergism, >10; indifference, −10 – 10; and antagonism, <−10 (68).

### Statistical analysis

All experiments were repeated at least two times with three replicates each unless otherwise noted. Data were expressed as the mean ± standard deviation. Statistical analysis was performed using R version 4.1.2 software. A two-tailed unpaired Student’s *t*-test was used to compare the two groups. *P*-values less than 0.05 were considered statistically significant (*P < 0.05, **P < 0.01, ***P < 0.001). For FHB disease control efficacy, data were subjected to one-way ANOVA, and the means of the treatments were separated by Duncan’s multiple range test (*P* < 0.05). Significant differences were indicated with different small letters in each bar.

## Data Availability

The transcriptome data sets were deposited in the National Center for Biotechnology Information Gene Expression Omnibus database with the accession number GSE214312.

## SUPPLEMENTAL MATERIAL

Supplemental material mbio.01351-24-s0001.pdf Fig. S1-S9, Tables S1-S3, and additional materials and methods.

ASM does not own the copyrights to Supplemental Material that may be linked to, or accessed through, an article. The authors have granted ASM a non-exclusive, world-wide license to publish the Supplemental Material files. Please contact the corresponding author directly for reuse.
