# Supplementary material for: Edeine B1 produced by Brevibacillus brevis reduces the virulence of a plant pathogenic fungus by inhibiting mitochondrial respiration
Source: mBio. 2024 Jun 11;15(7):e01351-24. doi: 10.1128/mbio.01351-24 (PMC11253638; doi:10.1128/mbio.01351-24)
Supplement: Supplemental material — Fig. S1-S9, Tables S1-S3, and additional materials and methods. [file mbio.01351-24-s0001.pdf]

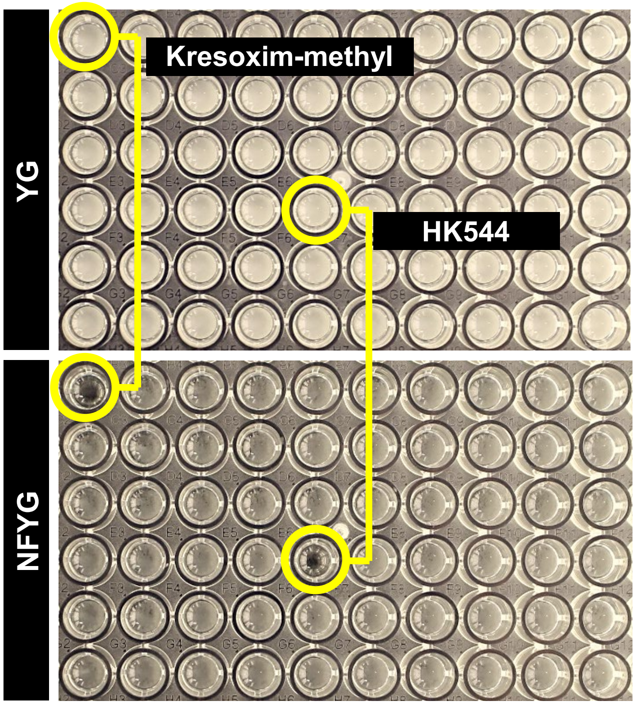

Fig S1. Effects of HK544 culture filtrate on growth of *Saccharomyces cerevisiae* in YG and NFYG media. Growth inhibition by representatives of fungal culture filtrates (20%, v/v). Photographs were taken one day after treatment. A QoI fungicide kresoxim-methyl was used as a positive control.

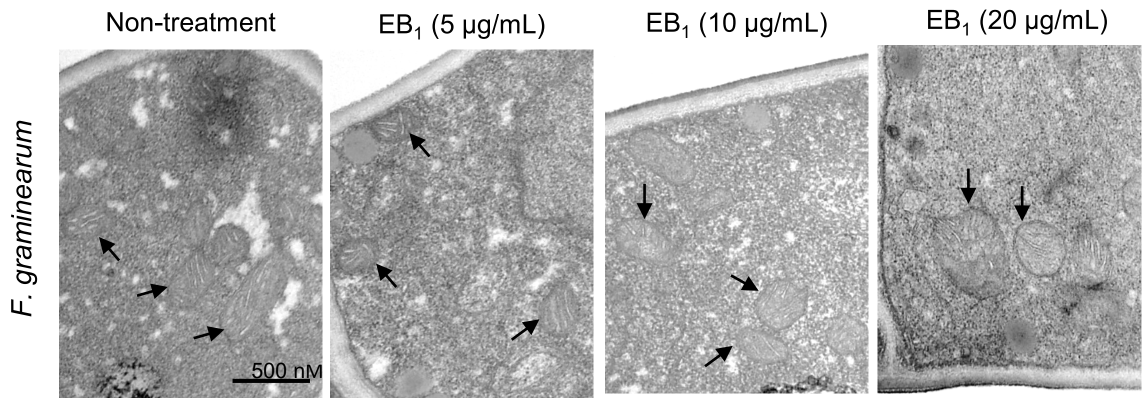

Fig S2. Transmission electron microscopy (TEM) observation of *Fusarium graminearum* treated with EB_1. TEM was conducted using *F. graminearum* conidia harvested after treatment with EB_1 for 4 h. Back arrows indicate mitochondria in *F. graminearum*.

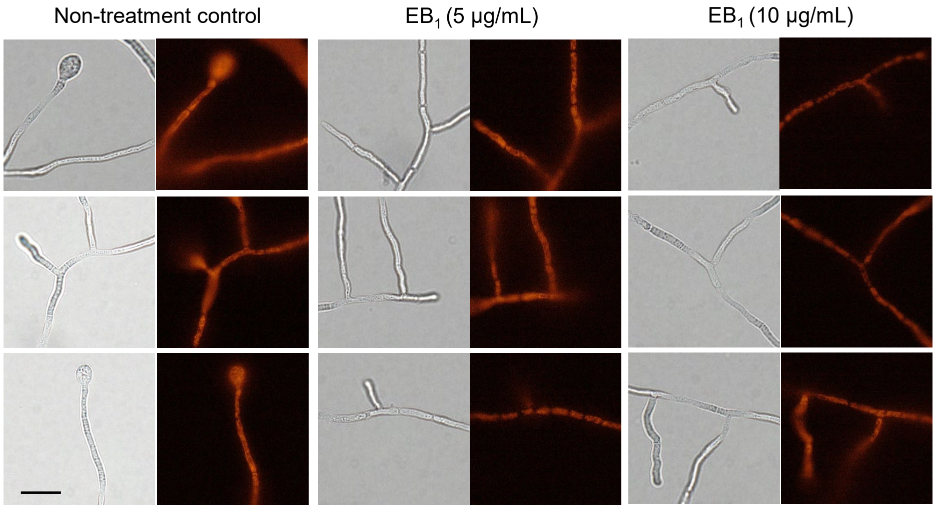

Fig S3. Visualization of mitochondrial superoxide generated in *Fusarium graminearum* treated with edeine B_1 (EB_1). The fungal mycelia were cultured in liquid CM with EB_1 for 4 h, and mitochondrial superoxide based on fluorescence intensity were investigated using MitoSOX Red. Scale bar = 20 μm.

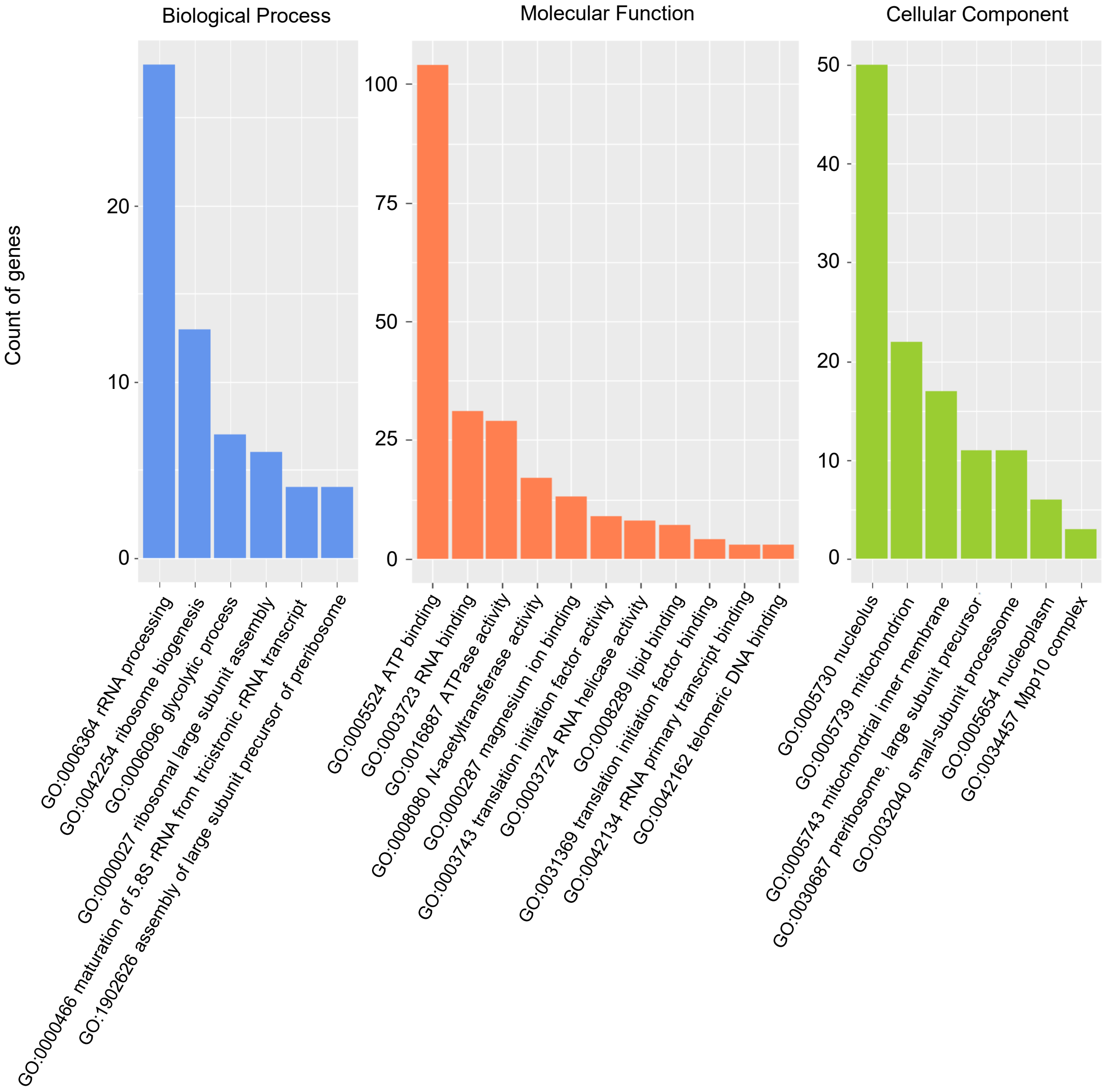

Fig S4. Gene ontology (GO) enrichment analysis of the DEGs. The GO terms for biological process (blue), cellular component (coral), and molecular function (green) each were determined with functional enrichment clustering analysis of 1,617 DEGs *(p* < 0.05). The values on the Y-axis indicate the number of genes detected in each GO term.

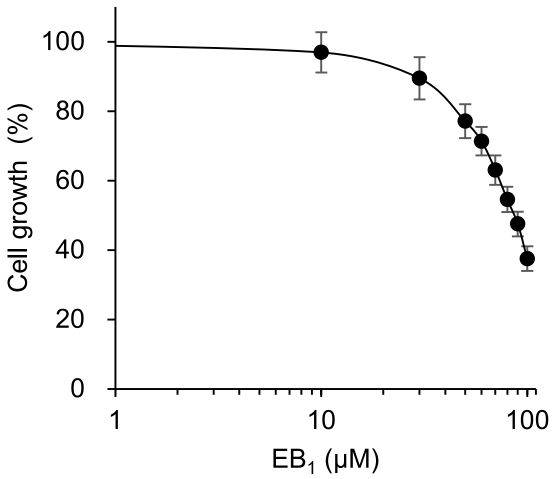

Fig S5. Determination of GI_{50} value of EB_1 against *Schizosaccharomyces pombe* SP286.

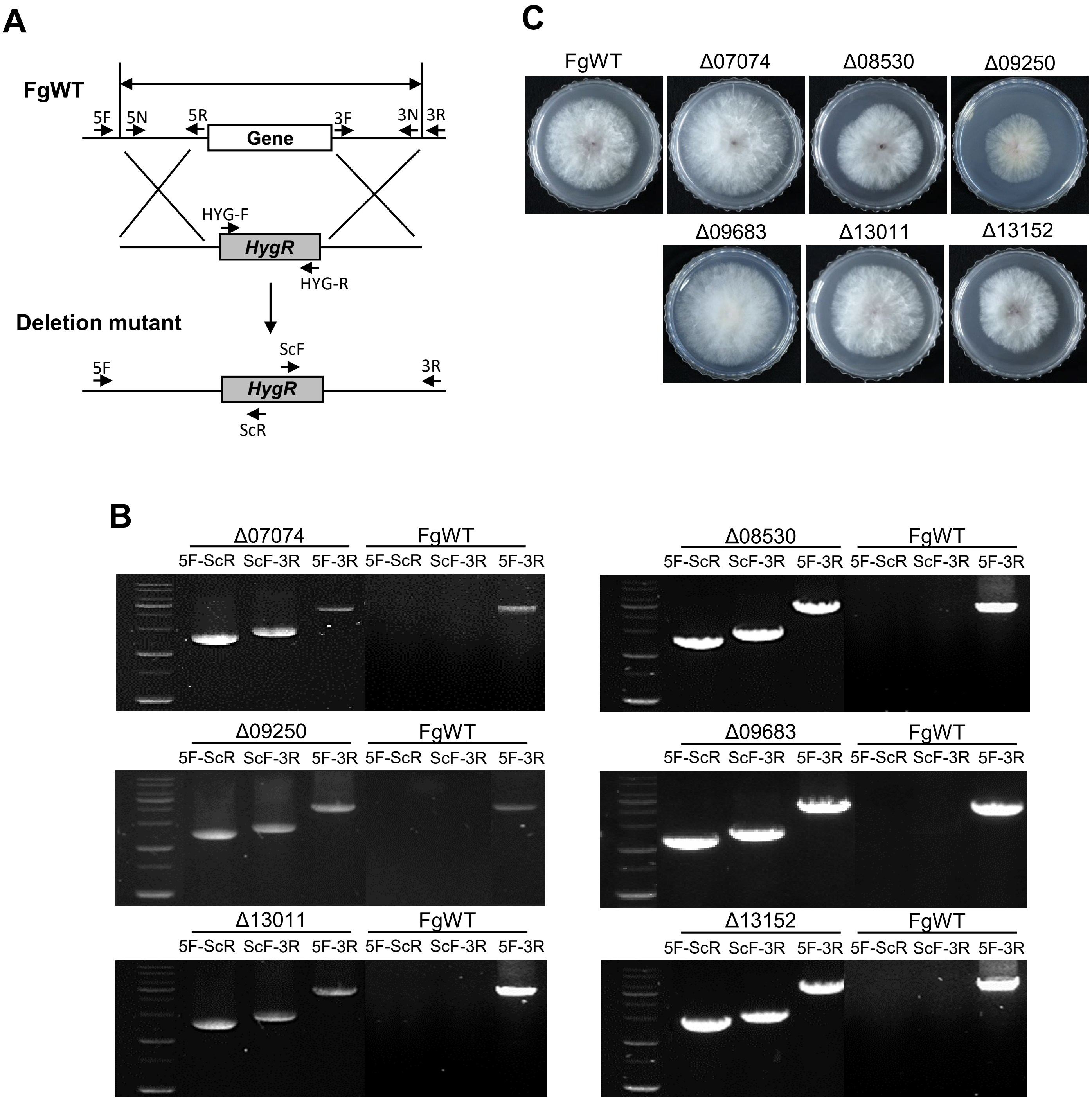

Fig S6. Strategies for target gene deletion in *Fusarium graminearum.* (A) Schematic representation for gene deletion by which the gene in *F. graminearum* wild-type strain was replaced with hygromycin resistance gene (*HygR*) by homologous recombination. (B) Confirmation of gene deletion by PCR. Three primer pairs were used, and the results showed distinct PCR amplifications between FgWT and deletion strains. (C) Vegetative growth of each strain on PDA medium.

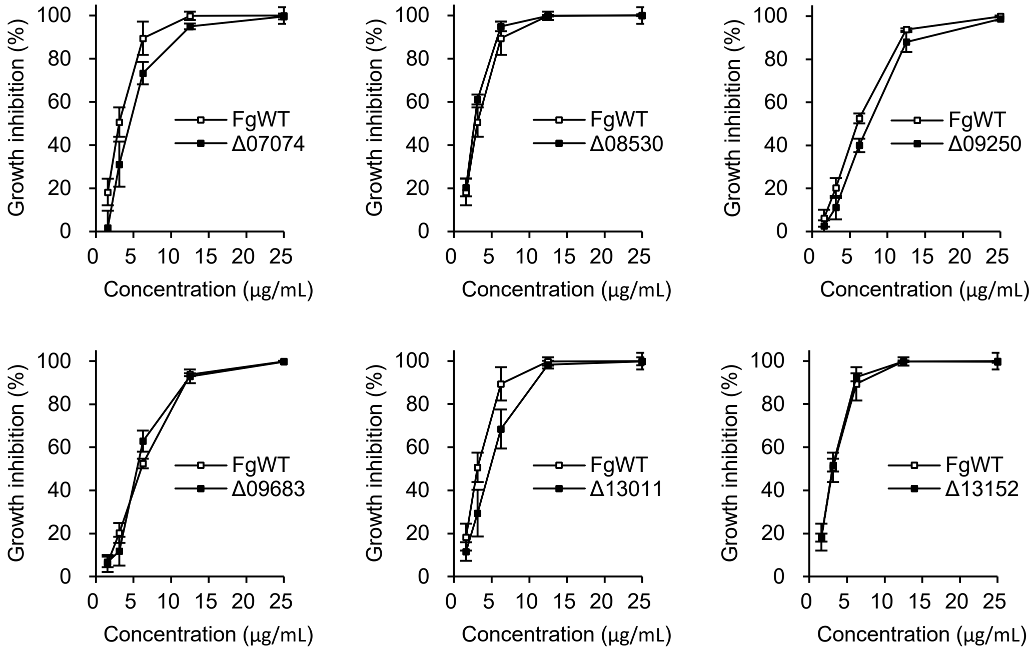

Fig S7. Comparison of growth inhibition between targeted-gene deletion strain and FgWT strain by EB_1 treatment. FgWT, *Fusarium graminearum* wild-type strain, Δ07074, an FGSG_07074 deletion strain; Δ08530, an FGSG_08530 deletion strain; Δ09250, an FGSG_09250 deletion strain; Δ09683, an FGSG_09683 deletion strain; Δ13011, an FGSG_13011 deletion strain; and Δ13152, an FGSG_13152 deletion strain.

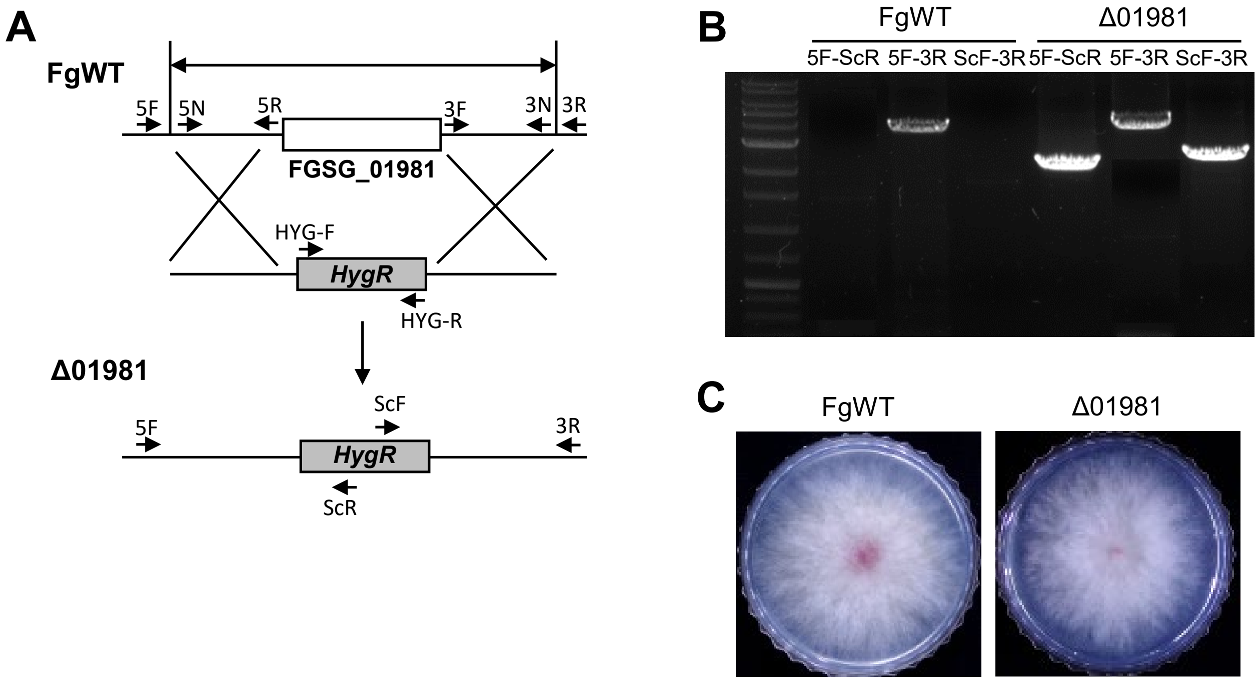

Fig S8. Strategies for deletion of FGSG_01981 in *Fusarium graminearum.* (A) Schematic representation for gene deletion by which the gene FGSG_01981 in *F. graminearum* wild-type strain was replaced with hygromycin resistance gene (*HygR*) by homologous recombination. (B) Confirmation of FGSG_01981 deletion by PCR. Three primer pairs were used, and the results showed distinct PCR amplifications between FgWT and Δ01981 strains. (C) Vegetative growth of each strain on PDA medium.

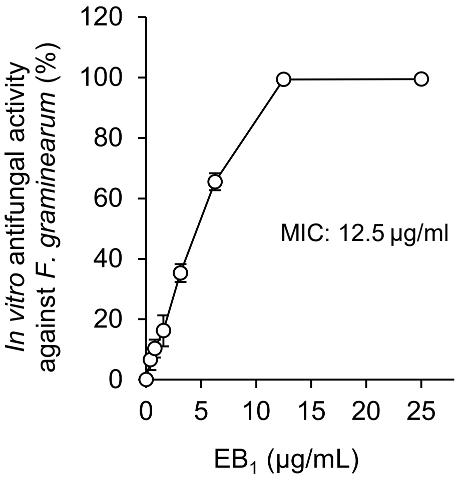

Fig S9. Minimum inhibitory concentration (MIC) of EB_1 against *Fusarium graminearum* using a conidial suspension (1×10^4 conidia/mL of PDB).

Table S1. The NMR assignments for of β-tyrosine, isoserine, α,β-diamino-propionic acid (DAPA), α-2,6-diamino-7-hydroxyazelaic acid (DAHAA), glycine, and polyamine of EB_1.

	Carbon	Chemical shift (ppm)	
		δ_C, type	δ_H (J in Hz)
β-Tyrosine	1	51.5, CH	4.64 dt (7.5, 3.9)
	2	56.4, CH_2	2.92 ddd (15.1, 8.2, 3.0); 3.0 m
	3	172.5, CO	
	1'	127.9, CH	
	2',6'	129.8, CH	8.55 d (8.5)
	3',5'	117, C	6.9 d (8.5)
	4'	157.8, C	
β-Serine	4	43.3, CH_2	3.27 dt (6.7, 2.0); 3.44 m
	5	71.2, CH	4.09 m
	6	175.9, CO	
DAPA	7	48.2, CH	4.71 dt (8.1, 4.0)
	8	170.3, CO	
	27	41.5, CH_2	3.34 dq (7.5, 3.7); 3.35 dq (14.5, 5.5)
DAHAA	9	52.8, CH	4.14 m
	10	32.1, CH_2	1.56 m; 1.69 m
	11	22.6, CH_2	1.26 m; 1.38 m
	12	27.6, CH_2	1.52 m; 1.76 m
	13	56.2, CH	3.27 dt (6.7, 2.0)
	14	68.5, CH	4.29 ddt (10.2, 6.9, 3.6)
	15	39.0, CH_2	2.42 m
	16	174.4, CO	
	28	179.4, CO	
Glycine	17	43.5, CH_2	3.87 d (2.5)
	18	172.8, CO	
Polyamine	19	37.1, CH_2	3.18 t (6.8)
	20	26.6, CH_2	1.60 m; 1.76 m
	21	40.1, CH_2	3.0 m
	22	43.7, CH_2	3.0 m
	23	26.1, CH_2	1.52 m; 1.60 m
	24	23.9, CH_2	1.52 m; 1.60 m
	25	46.0, CH_2	3.0 m
	26	157.5, C	

Table S2. The predicted binding free energies calculated from molecular dynamics (MD) simulations for the EB_1 to the cytosolic and mitochondrial ribosome

Target	Complex structure of ribosome binding to EB_1	Predicted ΔG of EB_1 binding (kcal/mol)
Cytosolic ribosome	X-ray	-60.13 ± 6.74
Mitochondrial ribosome	Docking model	-123.38 ± 9.12

Table S3. Primers used in this study

Primer	Sequence (5' to 3')	Purpose
Fg01981-5F	GGAGCAAGATGGACTCAACAT	Deletion for FGSG_01981
Fg01981-5R	tccactagctccagccaGGCAACGAGACCAACTCTTTA	
Fg01981-3F	cagaagaatagcttagcagagtctGTGTCATTGGTGGACTTGTATTG	
Fg01981-3R	TCTCATGCCGTTCCGTTATT	
Fg01981-5N	TCCATCTTCCCTGGTCACTA	
Fg01981-3N	AGCCATCATCCGCAAAGT	
Fg07074-5F	AGTCATGGTGAACGCCAAA	Deletion for FGSG_07074
Fg07074-5R	tccactagctccagccaGGTGATGCTGTTCCTGTTCT	
Fg07074-3F	cagaagaatagcttagcagagtctCGCCACACCACAAAGTCA	
Fg07074-3R	AAAGGCCGGCACCTATTT	
Fg07074-5N	GCTGAACGGAATAGGACATCA	
Fg07074-3N	CCTAGCTAACCAGGACTTGAAC	
Fg08385-5F	ACGGGCCTCTCGAAGAA	Deletion for FGSG_08385
Fg08385-5R	tccactagctccagccaCGGAGGTAGTCGCTACGAT	
Fg08385-3F	cagaagaatagcttagcagagtctCGGCGTCGTTTCCATATCTT	
Fg08385-3R	CCAAATGAGTACCCTCTTGAGC	
Fg08385-5N	GAGCCCATATCCGTACTTTGT	
Fg08385-3N	CTGAGCAGCTGGACCATATT	
Fg09250-5F	TTTGTTGGGAGAGCGATGG	Deletion for FGSG_09250
Fg09250-5R	tccactagctccagccaGACCGGGTGGTTCAGAAATAG	
Fg09250-3F	cagaagaatagcttagcagagtctCGTAAACTGCCCTCTCATACAA	
Fg09250-3R	GACTCATTACTGTCTCCCACAC	
Fg09250-5N	GTTTCACAGAGACCGCAGAA	
Fg09250-3N	TGCAATGACAACGTCCACT	
Fg09683-5F	GACTGGCTCGATTACCAGTTAAA	Deletion for FGSG_09683
Fg09683-5R	tccactagctccagccaGGAAGAATGCGGTGGAGAAA	
Fg09683-3F	cagaagaatagcttagcagagtctGTAGGAGATCTCTGGATAAATCAT	
Fg09683-3R	CCTGACTCTTCACAATCGTATTT	
Fg09683-5N	CTGCTTTCATGGAAACAACTAGAA	
Fg09683-3N	ACAACACCTGGGCAGTTT	
Fg13011-5F	ACCCAAGCATTCGGAAGAAG	Deletion for FGSG_13011
Fg13011-5R	tccactagctccagccaAGTTATGCGAGACCAAGTGATG	
Fg13011-3F	cagaagaatagcttagcagagtctACGACTGGAAATTTATGAGCATATC	
Fg13011-3R	TCAGCTTCGACCCATCATTAC	
Fg13011-5N	CTTTCCACCCATGCCTACA	
Fg13011-3N	GTCGATAAACTTCCGACTCCTC	
Fg13152-5F	CGTACGACAAGATACGGTGAT	Deletion for FGSG_13152
Fg13152-5R	tccactagctccagccaCTTGAAGACGTTGAGCAAAGC	
Fg13152-3F	cagaagaatagcttagcagagtctGGGTCATGTCCCACTAATTG	
Fg13152-3R	AACATGGCGGTTGGAAAG	
Fg13152-5N	GCTCAATGCCAAAGAGTTTCC	
Fg13152-3N	ACGACGAGCATCGATGAATAC	
HYG-F	TGGCTGGAGCTAGTGGA	Hygromycin resistance
HYG-R	AGACTCTGCTAAGCTATTCTTCTG	
ScF	CTGGACCGATGGCTGT	For the screening
ScR	TCCGAATGGGCCGAACC	

Table S3 (continued). Primers used in this study

Primer	Sequence (5' to 3')	Purpose
01981-rtF	CATCAACGGTATTCGTCACCT	
01981-rtR	TGTGGCTACTTCTGACTTCTTG	
00644-rtF	CAGCCAGGAGGATATCAAGAAG	
00644-rtR	CAAACAGGTCACCGACAGTA	
01941-rtF	GGAGGATGAGGGTTCTGAGTA	
01941-rtR	TCCCAGACCTTGGTCTTCT	
08888-rtF	CCACTGAACTCTGGAAGAAGAT	
08888-rtR	AGGAGGTCTTATCCTTGTTGTG	
09489-rtF	GAGGAAGAAGAGGAGGAGGAT	
09489-rtR	GTGGGCAAGGTGGAAGAA	For qRT-PCR
09547-rtF	CAGCGTCATCAAGGACATCA	
09547-rtR	AAAGGCTTGCCATCACCA	
09879-rtF	TTCGTCATGGCCAACTTCTT	
09879-rtR	GTTCTTCTGAGCAGCTCGTT	
10305-rtF	CCTTCAACGACGCCATCTT	
10305-rtR	GAGTTAAGGTACTCGCTTCGC	
Tri5-rtF	GACCCTAAGCGACTACAG	
Tri5-rtR	GTGCTACGGATAAGGTTC	
Tri6-rtF	GGCAACCATTCAAGCGCTTTTTCT	
Tri6-rtR	CACCCTGCTAAAGACCCTCAGACATT	
UHB-rtF	CAAGGTTACCGACAACAATA	
UHB-rtR	GCATCCATCAACTTCTTCAA	

Materials and Methods for Supplementary Information

Transmission electron microscopy

TEM was conducted using *F. graminearum* conidia harvested after treatment with EB_1 for 4 h. The harvested conidia fixed in 5% glutaraldehyde at room temperature for 1 h. The fixed samples were treated with 1% osmium for 1 h and dehydrated in an ethanol series (50%, 60%, 70%, 80%, 90%, and 100%) followed by drying using an dry oven. Polymerized samples were trimmed, sliced to a thickness of 80 nm, and observed under a Talos L120C (FEI, Czech) transmission electron microscope. The steps from trimming to TEM observation were kindly performed by the NICEM transmission electron microscope laboratory (Seoul National University, Seoul, Korea).

Observation of mitochondrial superoxide generation

To visualize mitochondrial superoxide generation, MitoSOX ™ Red (Invitrogen) was added to mycelia treated with EB_1 to a final concentration of 5 μM. After incubation for 20 min in the dark, the mycelia were washed twice with PBS. Microscopic observations were performed using by an Olympus BS53 microscope (Münster, Germany) with a consistent exposure time.

Molecular docking

A docking study was performed using the Schrödinger Suite 2024-1 (Schrödinger LLC, New York, USA, 2024) to predict the binding model of EB_1 against mitochondrial ribosome (PDB ID, 8OM4). The protein preparation was revised using Protein Preparation Wizard in Maestro v13.9. The receptor grid box for docking was generated with a 25 × 25 × 25 Å cubic size centered on the ATP binding site. The compound was minimized using an OPLS_2005 force field with a dielectric constant of 80.0 in MacroModel v14.3. The docking study of the EB_1 was performed using the standard precision method in Glide v10.2. The predicted binding model of the top 1 was selected for molecular dynamics (MD) simulations.

Molecular dynamics (MD) simulation

The MD simulations with an explicit solvent model were performed using Desmond v7.7 with OPLS4 force field (Desmond Molecular Dynamics System; D. E. Shaw Research: New York, NY, 2024). The initial structure of the cytosolic ribosome bound to EB_1 was adapted from the X-Ray co-crystal structure from the PDB bank (PDB ID, 4U4N), and mitochondrial ribosome bound to EB_1 was a result from the molecular docking study. System Builder was used for solvation, employing predefined TIP3P water in an orthorhombic box with dimensions of 25 Å × 25 Å × 30 Å. The overall complex structure was neutralized by adding Cl^- counterions. The NaCl salt concentration was 0.15 mol/L. Then, production MD simulations of 100 ns in length were carried out under periodic boundary conditions in the NPT ensemble at normal temperature (300 K) and pressure (1.01325 bar) with the default setting of relaxation before simulation. Recording intervals of 1.2 and 100 ps were used for energy calculation and trajectory analysis.

Molecular mechanics-generalized Born surface area (MM-GBSA) calculation

The final equilibrium state for protein-compound complexes were rescored with the MM-GBSA approach, as implemented in the Prime MM-GBSA module in the Schrödinger Suite. The binding free energy was calculated for 20 frames based on the last 100 ns MD trajectory.
